# An ultra-early, transient interferon-associated innate immune response associates with protection from SARS-CoV-2 infection despite exposure

**DOI:** 10.1016/j.ebiom.2024.105475

**Published:** 2024-12-11

**Authors:** Joe Fenn, Kieran Madon, Emily Conibear, Romain Derelle, Sean Nevin, Rhia Kundu, Seran Hakki, John S. Tregoning, Aleksandra Koycheva, Nieves Derqui, Mica Tolosa-Wright, Jakob Jonnerby, Lulu Wang, Samuel Baldwin, Timesh D. Pillay, Ryan S. Thwaites, Constanta Luca, Robert Varro, Anjna Badhan, Eleanor Parker, Carolina Rosadas, Myra McClure, Richard Tedder, Graham Taylor, Ajit Lalvani, INSTINCT Study Investigators, Joe Fenn, Kieran Madon, Emily Conibear, Romain Derelle, Sean Nevin, Rhia Kundu, Seran Hakki, Aleksandra Koycheva, Nieves Derqui, Mica Tolosa-Wright, Jakob Jonnerby, Lulu Wang, Samuel Baldwin, Timesh Pillay, Ryan Thwaites, Constanta Luca, Robert Varro, Anjna Badhan, Eleanor Parker, Carolina Rosadas, Myra McClure, Richard Tedder, Graham Taylor, Ajit Lalvani, Janakan Narean, Lucy Mosscrop, Patricia Watber, Jie Zhou, Jack Barnett, Hamish Houston, Anika Singanayagam, Paul Freemont, Neil Ferguson, Maria Zambon, Wendy Barclay, Jake Dunning, Jessica Cutajar, Valerie Quinn, Sarah Hammett, Eimèar McDermott, Kristel Timcang, Jada Samuel, Samuel Bremang, Samuel Evetts, Megan Davies, Chitra Tejpal, Anjeli Ketkar, Giulia Miserocchi, Harriet Catchpole, Simon Dustan, Isaac Day Weber, Federica Marchesin, Alexandra Kondratiuk

**Affiliations:** a NIHR Health Protection Research Unit in Respiratory Infections, Imperial College London, London, UK; b National Heart and Lung Institute, Imperial College London, London, UK; c Department of Infectious Disease, Imperial College London, London, UK; d MRC Centre for Global Infectious Disease Analysis, School of Public Health, Imperial College London, London, UK

**Keywords:** SARS-CoV-2, Resistance to infection, Household contacts, Innate immunology

## Abstract

**Background:**

A proportion of individuals exposed to respiratory viruses avoid contracting detectable infection. We tested the hypothesis that early innate immune responses associate with resistance to detectable infection in close contacts of COVID-19 cases.

**Methods:**

48 recently-exposed household contacts of symptomatic COVID-19 cases were recruited in London, UK between May 2020 and March 2021 through a prospective, longitudinal observational study. Blood and nose and throat swabs were collected during the acute period of index case viral shedding and longitudinally thereafter. Magnitude of SARS-CoV-2 exposure was quantified, and serial PCR and serological assays used to determine infection status of contacts. Whole-blood RNA-seq was performed and analysed to identify transcriptomic signatures of early infection and resistance to infection.

**Findings:**

24 highly-exposed household contacts became PCR-positive and seropositive whilst 24 remained persistently PCR-negative and seronegative. A 96-gene transcriptomic signature of early SARS-CoV-2 infection was identified using RNA-seq of longitudinal blood samples from PCR-positive contacts. This signature was dominated by interferon-associated genes and expression correlated positively with viral load. Elevated expression of this 96-gene signature was also observed during exposure in 25% (6/24) of persistently PCR-negative, seronegative contacts. PCR-negative contacts with elevated signature expression had higher-magnitude SARS-CoV-2 exposure compared to those with low signature expression. We validated this signature in SARS-CoV-2-infected individuals in two independent cohorts. In naturally-exposed healthcare workers (HCWs) we found that 7/58 (12%) PCR-negative HCWs exhibited elevated signature expression. Comparing gene-signature expression in SARS-CoV-2 Controlled Human Infection Model (CHIM) volunteers pre- and post-inoculation, we observed that 14 signature genes were transiently upregulated as soon as 6 hr post-inoculation in PCR-negative volunteers, while in PCR-positive volunteers gene-signature upregulation did not occur until 3 days later.

**Interpretation:**

Our interferon-associated signature of early SARS-CoV-2 infection characterises a subgroup of exposed, uninfected contacts in three independent cohorts who may have successfully aborted infection prior to induction of adaptive immunity. The earlier transient upregulation of signature genes in PCR-negative compared to PCR-positive CHIM volunteers suggests that ultra-early interferon-associated innate immune responses correlate with, and may contribute to, protection against SARS-CoV-2 infection.

**Funding:**

This work was supported by the 10.13039/100018336NIHR Health Protection Research Unit in Respiratory Infections, United Kingdom, 10.13039/501100000761NIHR Imperial College London, United Kingdom (Grant number: NIHR200927; AL) in partnership with the UK Health Security Agency and the 10.13039/501100000265NIHR Medical Research Council (MRC), United Kingdom (Grant number: MR/X004058/1). Support for sequencing was provided by the Imperial BRC Genomics Facility which is funded by the 10.13039/501100000272NIHR, United Kingdom. The development of the hybrid DABA assay used for quantification of SARS-CoV-2 anti-Spike RBD antibodies was supported by the 10.13039/501100000265MRC (MC_PC_19078).

**Research in context**

**Evidence before this study**

Meta-analyses of COVID-19 household contact studies show that a large proportion of unvaccinated contacts do not become infected by SARS-CoV-2 despite being highly exposed. Similarly, the only published SARS-CoV-2 controlled human infection model (CHIM) study in unvaccinated individuals showed that 17/36 (47%) of participants resisted infection despite direct intranasal inoculation, consistent with natural immune resistance to infection in a proportion of individuals. We searched PubMed to review evidence of immune mechanisms of protection from SARS-CoV-2 infection using the search term ‘SARS-CoV-2 AND cohort AND (Immune OR immunological OR innate OR adaptive) AND (protection OR resistance OR seronegative) NOT vaccination’. Two publications suggested that pre-existing cross-reactive T cells have a role in mediating protection from infection in seronegative individuals. One found higher frequencies of cross-reactive T cells in highly exposed uninfected household contacts of COVID-19 cases compared to infected contacts at the time of exposure. Another observed expansion of cross-reactive T cells in some highly exposed but uninfected individuals. Although pre-existing T cells likely play an important role, their presence was not evident in the majority of uninfected contacts investigated in these studies. Given that most exposed uninfected contacts do not harbour detectable pre-existing cross-reactive T cells, other mechanisms must play a role in mediating protection against infection. We found no publications in which immune factors other than pre-existing cross-reactive T cells associated with resistance to infection.

**Added value of this study**

We recruited a unique cohort of close household contacts of COVID-19 cases and collected samples during exposure, and longitudinally thereafter, to investigate innate immune correlates of early SARS-CoV-2 infection and protection from infection. We identified a dynamic immune signature of early SARS-CoV-2 infection which was dominated by interferon (IFN) signalling-associated genes and correlated positively with upper respiratory tract viral load. We demonstrated that 6/24 (25%) of persistently PCR-negative and seronegative contacts also exhibited upregulation of this IFN-associated gene signature following SARS-CoV-2 exposure, consistent with innate immune responses mediating protection from infection. We validated our finding using two additional, independent SARS-CoV-2-exposed cohorts including persistently PCR-negative healthcare workers and volunteers challenged by direct intranasal SARS-CoV-2 inoculation. In the CHIM data, we saw transient upregulation of signature genes 6 hr post-inoculation in participants who remained uninfected, but not in those who went on to become infected. The added value of this study was enabled by the rigorous clinical and epidemiological phenotyping of our cohort combined with longitudinal collection of samples starting during acute SARS-CoV-2 exposure. As such, our study design, combined with the frequent, early post-inoculation sampling in the CHIM, represents a blueprint for future studies aiming to identify immune correlates of protection from viral infection.

**Implications of all the available evidence**

We show that expression of a transcriptomic signature of early SARS-CoV-2 infection is elevated in some persistently PCR-negative, seronegative contacts of COVID-19 cases during exposure. Signature expression, alongside persistent PCR negativity and seronegativity, defines a previously overlooked phenotype of effective abrogation of infection following exposure. The genes comprising this transcriptomic signature are associated with interferon response pathways, compatible with a role in protection from infection. The ultra-early transient upregulation of genes within this signature post-SARS-CoV-2 inoculation in persistently PCR-negative, but not PCR-positive, volunteers identifies a correlate and potential mechanism of protection against sustained infection. Our findings, in the context of existing literature, support a multifactorial model of immune resistance to SARS-CoV-2 infection involving both pre-existing cross reactive T cells (as previously reported) and innate immune mechanisms (as identified here). Moreover, our findings challenge the paradigm that seroconversion is synonymous with infection, revealing that early innate immune responses have the potential to abort transient infection without induction of a measurable antibody response. This evidence suggesting innate immune protection from infection despite high-magnitude exposure supports development of future prophylactics designed to leverage these innate pathways to prevent infection.

## Introduction

A substantial proportion of individuals exposed to respiratory viruses do not become infected, including close household contacts with intense exposure.^1^^,^^2^ The observation, early during the COVID-19 pandemic, that a proportion of antigen-naïve SARS-CoV-2-exposed individuals resisted infection suggested that early innate immune mechanisms might play an important role in resistance to acquisition of infection.^1^^,^^3^^,^^4^ Frequent breakthrough infections in vaccinated and previously infected individuals also highlight the limited protection from infection afforded by adaptive immunity.^5^ These observations make delineating innate immune mechanisms of protection from SARS-CoV-2 infection, which could be leveraged to confer broad protection against a range of variants and viruses, a public health priority.

Defining the innate immune responses that mediate natural resistance to infection is challenging, not least because of the partially protective effects of acquired adaptive humoural and cellular immune responses.^6^ In order to optimally identify innate correlates of protection, whilst limiting the potentially confounding effects of adaptive immune responses, it is necessary to recruit cohorts that meet a number of criteria. Firstly, samples must be collected from antigen-naïve individuals who have had significant, well-defined, exposure to the pathogen. Secondly, samples must be collected during or immediately after acute pathogen exposure, while innate immune responses mediating resistance to infection are ongoing. Thirdly, the absence of measurable infection despite exposure must be reliably confirmed by longitudinally testing for evidence of infection by both PCR and serologically.

We leveraged the unique natural experiment of the COVID-19 pandemic to recruit a prospective, longitudinal cohort of unvaccinated, SARS-CoV-2-naïve household contacts of newly diagnosed PCR-confirmed COVID-19 index cases between May 2020 and March 2021, who fulfilled the above criteria to delineate immune correlates of protection from SARS-CoV-2 infection.^1^^,^^3^^,^^7^ Rigorous clinical and epidemiological phenotyping was performed and biological samples were collected from contacts at enrolment, during ongoing index cases viral shedding, and serially thereafter on days 4, 7, 14, and 28 post-enrolment. This enabled longitudinal PCR and serological testing to exclude sustained, replicative infection. Global transcriptomic responses were assessed by genome-wide RNA-seq on serial blood samples from PCR-positive and highly exposed but persistently PCR-negative household contacts, as well as unexposed controls.

We tested the hypothesis that early innate immune responses associate with protection from PCR or serologically defined SARS-CoV-2 infection. We first discovered transcriptomic signatures of early SARS-CoV-2 infection in PCR-positive contacts and subsequently investigated whether similar signatures were evident in persistently PCR-negative seronegative contacts with ongoing exposure. Furthermore, we investigated whether additional genes were differentially expressed between exposed PCR-negative contacts and unexposed controls. Finally, we validated our newly identified transcriptomic signature of recent SARS-CoV-2 exposure in two independent cohorts: occupationally-exposed SARS-CoV-2-naïve healthcare workers and healthy volunteers intranasally inoculated in the SARS-CoV-2 controlled human infection model (CHIM). In the latter, we also compared the dynamics of gene-signature expression pre- and post-SARS-CoV-2 inoculation between PCR-negative and PCR-positive participants.

## Methods

### Study design

From May 2020 to March 2021, SARS-CoV-2 infected index cases and their household contacts were referred to the INSTINCT (Integrated Network for Surveillance, Trials and Investigations into COVID-19 Transmission) study team via the National Health Service and UK Health Security Agency national test and trace scheme having provided informed consent to participate (REC Reference: 20/NW/0231 IRAS: 282820). Contacts of index cases that were referred to the INSTINCT study team within 5 days of index symptom onset were enrolled onto the study. Research nurses visited study participants on the day of enrolment (day 0) and days 7, 14, and 28 post enrolment. During study visits, blood and nose and throat swabs were collected. An additional self-collected nose and throat swab was collected on day 4. Demographic information was collected following enrolment and is presented in Table 1. All demographics, including sex, were self-reported by participants.

**Table 1:** Cohort demographic data.

	Total (N = 48)	PCR-positive (N = 24)	PCR-negative (N = 24)	p-Value
**Sex**				0.0155
Female	24 (50.0%)	7 (29.2%)	17 (70.8%)	
Male	24 (50.0%)	17 (70.8%)	7 (29.2%)	
**Age (years)**				0.587
<18	6 (12.5%)	4 (16.7%)	2 (8.3%)	
18–39	17 (35.4%)	8 (33.3%)	9 (37.5%)	
40–59	21 (43.8%)	9 (37.5%)	12 (50.0%)	
≥60	4 (8.3%)	3 (12.5%)	1 (4.2%)	
**BMI**^a^				0.0405
Normal weight	18 (37.5%)	3 (12.5%)	15 (62.5%)	
Overweight	17 (35.4%)	11 (45.8%)	6 (25.0%)	
Obese	4 (8.3%)	3 (12.5%)	1 (4.2%)	
Missing	9 (18.8%)	7 (29.2%)	2 (8.3%)	
**Ethnicity**				0.933
White	41 (85.4%)	20 (83.3%)	21 (87.5%)	
Non-White	5 (10.4%)	2 (8.3%)	3 (12.5%)	
Missing	2 (4.2%)	2 (8.3%)	0 (0%)	
**Comorbidities**^b^				0.471
Y	7 (14.6%)	2 (8.3%)	5 (20.8%)	
N	41 (85.4%)	22 (91.7%)	19 (79.2%)	

Demographic characteristics of PCR-positive and PCR-negative contacts. p Values of χ^2^ tests used to compare groups are displayed.

a BMI thresholds are as follows: normal weight ≥18.5 & <25; overweight ≥25 & <30; and obese ≥30. BMI is excluded for individuals under the age of 18.

b Comorbidities include three cases of asthma and one case of each of the following: high blood pressure, hypothyroidism, chronic cardiac disease, and diabetes.

### Cohort selection

From a total of 383 individuals recruited to the INSTINCT study, 48 household contacts were included in this study as they fulfilled the following inclusion criteria: i) they were household contacts of a SARS-CoV-2 confirmed index case; ii) they had PCR and serology data available for 2 or more timepoints; iii) they had no history of COVID-19 vaccination or infection determined by medical records and/or serology; iv) they were not hospitalised as a result of SARS-CoV-2 infection; v) they had available exposure information (see below); vi) they had available PAXgene samples from 2 or more study timepoints (including day 0 and 7). Contacts were assigned to the PCR-positive group if they had confirmed SARS-CoV-2 infection during the study (defined as a PCR-positive result from the nose and throat swabs at one or more study days). Contacts were assigned to the PCR-negative group if they remained persistently PCR-negative and seronegative throughout the study, and had a high level of exposure to an index case, defined as sharing living space with a PCR-positive index case and having an exposure score >6000 (see below for calculation used to derive exposure score). Visualisation of the full cohort breakdown can be found in Figure S1.

14 unexposed healthy controls were also included in this study. All unexposed healthy controls had samples collected at a single timepoint. 8 of these were asymptomatic, persistently PCR-negative and seronegative individuals without any known exposure to COVID-19 index cases, recruited before August 2020 as part of the INSTINCT study. 6 were pre-pandemic healthy controls collected as part of the HPRU in Respiratory Infections Research Tissue Bank (REC Reference: 07/H0712/85).

### RT-PCR quantification of viral load

Nose and throat samples were collected using flocked swabs placed in COPAN Universal Transport Medium (Copan Diagnostics, Murrieta, CA, USA) in participant's homes. RT-PCR was performed as previously described using TaqMan Fast Virus 1-Step Master Mix (Thermo Fisher Scientific, Waltham, MA, USA) in a duplex PCR targeting viral E and host RNAse P.^8^ Samples with adequate RNAseP RNA and an E gene Ct <36.5 (which equates to 5 RNA copies per PCR reaction) were considered PCR-positive.

### Hybrid double antigen binding assay (DABA)

Total antibodies specific for SARS-CoV-2 RBD were quantified by a highly sensitive and specific double antigen binding assay, as described previously.^9^ Briefly, solid phase 96-microwells plates were coated with 100 μl of S1 antigen at a concentration of 5 μg/ml. Control and test sera were added to washed plates and incubated for 1 h at 37 °C. Detection was performed with RBD-HRP and development with TMB and plates read spectrometrically at 450–630 nm. A DABA binding ratio result of 1 (AU) was used as the threshold for seropositivity.

### SARS-CoV-2 exposure quantification

The extent of contact exposure to PCR-confirmed index cases was calculated based on information provided by participants in detailed Case Record Forms using the following formula:

$$Exposure\;score=Relationship\;Score*Room\;Share\;Score*\mathrm{Log}\;10\;\left(Index\;viral\;load\;AUC\right)$$

Relationship score describes the relationship of the contact to the index case with values of 100, 80, or 60 being given to individuals in romantic partnerships, parent/child or sibling relationships and other cohabitant relationships respectively.

Room share score describes the level of room sharing between contact and index throughout the house. Individuals who shared a bedroom with the index case were given a score of 100, individuals which did not share a bedroom but did share a bathroom were given a score of 80, and individuals who did not share a bedroom or bathroom, but did share other living spaces (e.g., kitchen or living room) with the index case were given a score of 60.

Log10 (Index viral load AUC) describes the area under the curve quantification of viral load copy number of the index. These values were estimated using the minimum area under the curve trapezoidal rule method, as previously described.^10^

Alongside quantifying the magnitude of exposure, behavioural changes that occurred between the contact and index cases were also documented at the time of enrolment. Behavioural changes included using different rooms, self-isolating from the index case or increasing the frequency of cleaning surfaces and/or hands.

Hand swab and environmental air samples were collected to test for the presence of SARS-CoV-2, as previously described.^1^ In brief, both hands of the index cases were swabbed and subjected to SARS-CoV-2 specific RT-PCR. For air sampling, air was collected from the most frequently shared area of the household using a Coriolis micro-biological air sampler. SARS-CoV-2 RNA was detected using AgPath-ID One-Step RT-PCR Reagents with specific primers and probes targeting the envelope and ORF1a genes.

### RNA-seq

2.5 ml of blood was collected in PAXgene tubes and stored at −20 °C within 6 hours of receipt, before being transferred to −80 °C. Total RNA was extracted from whole blood using the PreAnalytiX PAXgene Blood RNA Kit as per manufacturer's instructions and subsequently stored at −80 °C. Quantity, quality and integrity of RNA was determined used NanoDrop and Bioanalyser (RNA Nano 6000 Chip, Agilent) respectively. All sequenced samples had a RIN above 8. Library preparation and mRNA-sequencing was performed at Imperial BRC Genomic Facility. In brief, total RNA underwent poly(A) selection, globin and rRNA depletion and DNA libraries were constructed using the NEBNext® Ultra™ II Directional RNA Library Prep Kit for Illumina (New England Biolabs). These were then sequenced on the Illumina HiSeq 4000 using the HiSeq 3000/4000 PE Cluster and SBS kits (Illumina) producing a median of 26.1 million (range 18.34–56.04) 75bp paired-end reads per sample.

Raw data was converted into FASTQ files using bcl2fastq (Illumina). Raw FASTQ files were then processed with ‘Trim Galore!’ (https://www.bioinformatics.babraham.ac.uk/projects/trim_galore/) to remove adaptor sequences and low-quality reads. Samples were quality controlled using FastQC and MultiQC (https://www.bioinformatics.babraham.ac.uk/projects/fastqc/).^11^ Fastq files were aligned to the human reference genome (NCBI Human GRCh38.p13) using STAR aligner (version 2.7.1a). 144 blood RNA samples from 62 individuals gave a median of 14.26 million (range 7.30–37.67) mapped reads per sample. Read count matrices were generated using featureCounts from the Rsubread package.

Despite globin depletion during library preparation, we still observed substantial read counts for globin genes, as previously described.^12^ Therefore, we removed counts from the following globin-associated genes: *CYGB*, *HBA1*, *HBA2*, *HBB*, *HBD*, *HBE1*, *HBG1*, *HBG2*, *HBM*, *HBQ1*, *HBZ*, and *MB*. The following rRNA genes were also removed prior to downstream analysis: *RNA45SN1*, *RNA45SN2*, *RNA45SN3*, *RNA45SN4*, and *RNA45SN5*. Genes which were not present in all samples or if their variance across all samples was below 1 × 10^−10^ were also removed. Alternatively spliced genes were removed by retaining the copy with the highest mean across all samples.

The full analytical pipeline is represented graphically in Figure S2. Differential gene expression analysis was performed in R using DESeq2 with read counts being normalised using variance-stabilising transformation prior to principal component analyses and hierarchical clustering. All hierarchical clustering was performed on a Z-scored matrix, with Ward linkage and Euclidean distancing to generate both sample and gene clusters.

### Contribution to total variance (CTV) analysis

To investigate diversity between groups of samples without an *a priori* definition of comparison groups, CTV analysis was performed. This method, based on species contribution to β-diversity used in ecological studies to identify species that contribute the most to dissimilarity in a community,^13^ was adapted to identify genes that contribute most to the variance across a given dataset. Briefly, normalised gene read counts were transformed using the Hellinger transformation to yield CTV values which estimate feature contributions to the overall signal. Genes were sorted by CTV score, facilitating extraction of the most contributing features from a large dataset. Full methods and Python script available at: https://github.com/rderelle/SCBD.

### Discordance and Concordance (DISCO) analysis of differential gene expression

DISCO analysis was used to measure the level of concordance in the outputs of two differential gene expression analyses.^14^^,^^15^ Log2 fold change and associated p-values of differentially expressed genes (DEGs) derived from differential gene expression analyses were used to calculate DISCO scores for each gene, using the below equation:

$$disco.score=\log_{2}{FC}_{1}\;\times \;\log_{2}{FC}_{2}\;\times \;\left|\left(\log_{10}\left({pval}_{1}\right)\;+\;\log_{10}\left({pval}_{2}\right)\right)\right|$$

DISCO scores were scaled using a diverging log scale. A constant of 1 was added to the absolute value of the DISCO scores, and the natural log of the corrected value was plotted.

$$\log\;\text{transformed}\;\text{score}=\pm \ln\;\left(|\left(disco.score\right)|\;+\;1\right)$$

To account for converting negative input values to absolute values, scaled values derived from negative raw DISCO scores were converted to equivalent negative values after log transformation to maintain the separation of discordant and concordant DISCO scores.

### Validation datasets

The first validation dataset represented in Fig. 5 was acquired from the COVIDsortium observational cohort study in healthcare workers (HCW; NCT04318314) described in detail in Gupta et al.^16^ Briefly, blood and nasopharyngeal swabs were collected weekly from HCWs from St Bartholomew's Hospital (London, UK) and subject to whole-blood RNA-seq and SARS-CoV-2 RT-PCR testing. RNA-seq was performed as previously described using an Illumina Nextseq 500/550 High Output 75 cycle kit giving a median of 26 million reads per sample.^16^^,^^17^ Read count matrices and associated metadata were obtained from ArrayExpress (E-MTAB-10022).

The second validation dataset contained data from a SARS-CoV-2 controlled human infection model (CHIM). Here, 34 healthy individuals were inoculated with 10 TCID_50_ wild type SARS-CoV-2 intranasally.^18^^,^^19^ Blood and nasopharyngeal swabs were collected at 6 hr post inoculation and then on day's 1–5, 7, 10, 14, and 28 post inoculation. 18 individuals became PCR-positive whilst 16 individuals resisted infection and remained persistently PCR-negative and seronegative. RNA-seq was performed as previously described using the Illumina NovaSeq 6000 platform and NovaSeq 6000 S4 Reagent Kits (200 cycles).^18^ A median of 69.3 million 100 basepair paired-end reads were obtained per sample. Read count matrices and metadata were downloaded from Array Express (E-MTAB-12993). Data from 2 participants was excluded from the analyses as they were seropositive at the time of inoculation.

### Statistical analyses

Statistical analyses were performed using GraphPad Prism version 9, R version 4.1.0 and Python version 3. All visualisations of RNA-seq data were created in R using ggplot2 and pheatmap packages or GraphPad Prism with full description of statistical tests found in corresponding figure or table legends. Log fold change and adjusted p value cut offs for DEGs were defined in the relevant Results section. The threshold for genes which contributed to the variance across the dataset was a contribution score of >0.0002. GO analysis was conducted in ShinyGo v0.77 using GO Biological Processes as the reference pathway database.

### Ethics

Ethical approval for this study was obtained from the Northwest - Greater Manchester East Research Ethics Committee in accordance with Health Research Authority regulations (REC Reference: 20/NW/0231 IRAS: 282820). All participants were fully informed about the study's objectives, procedures, potential risks, and benefits and provided written informed consent prior to their inclusion in the study.

### Role of funders

This work is supported by the NIHR Health Protection Research Unit in Respiratory Infections, Imperial College London in partnership with the UK Health Security Agency (Grant number: NIHR200927; AL) and Medical Research Council (Grant number: MR/X004058/1). The Imperial BRC Genomics Facility is supported by NIHR funding to the Imperial Biomedical Research Centre. The development of the hybrid DABA assay used for quantification of SARS-CoV-2 anti-Spike RBD antibodies was supported by MRC grant: MC_PC_19078: nCoV: Serological detection of past SARS-CoV-2 infection by non-invasive sampling for field epidemiology and quantitative antibody detection. Study funders had no role in study design, data collection, data analyses, interpretation, or writing of this report.

## Results

### Prospective longitudinal COVID-19 household contact study design

**Fig. 1:**
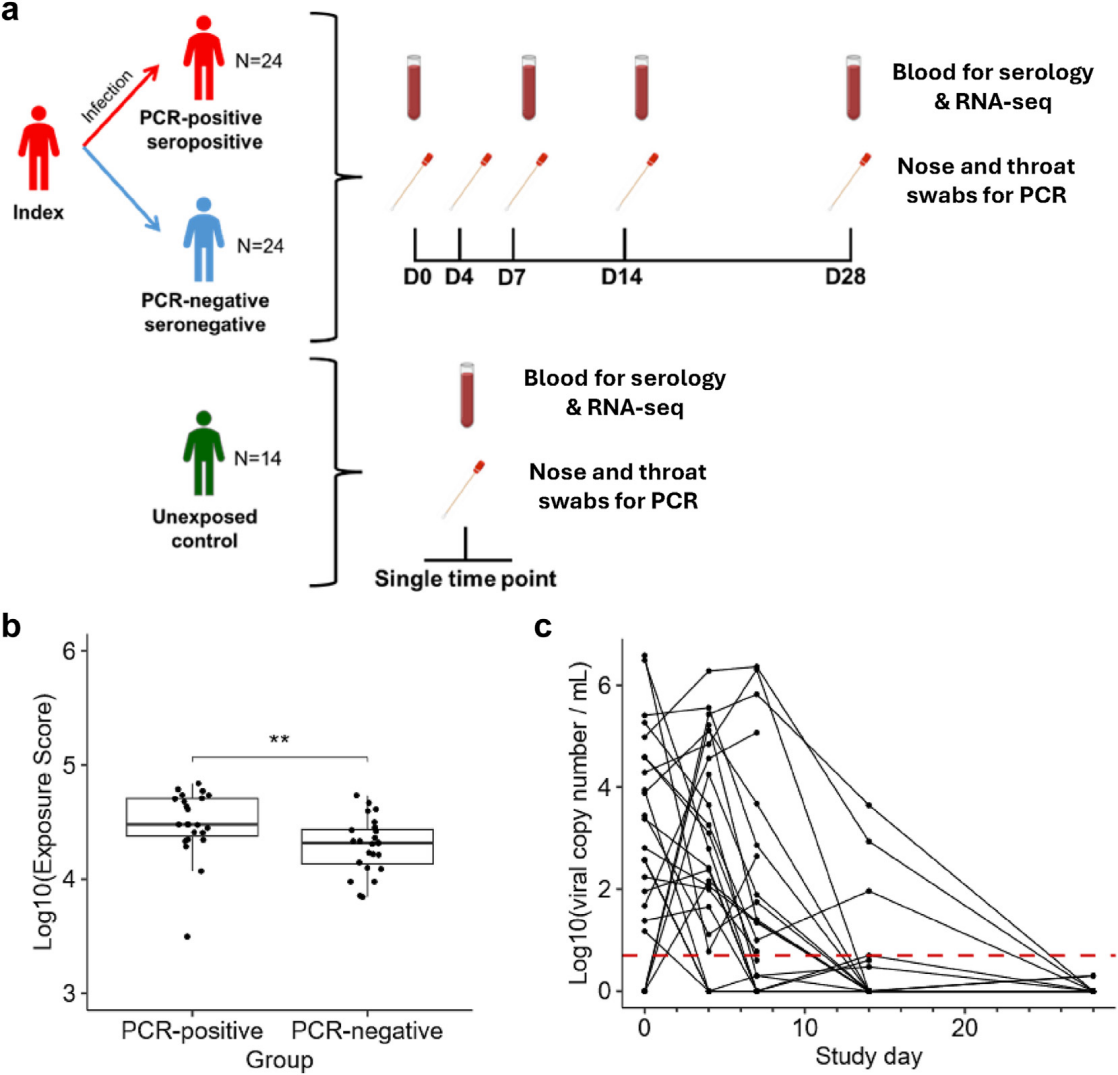
**Study cohort and sampling schedule.** (**a**) 48 household contacts of COVID-19 cases and 14 unexposed controls were recruited. Serial blood and nose and throat swab samples were collected at study day 0 (D0), and days 7, 14, and 28 post enrolment (D7, D14, and D28 respectively). RNA-Seq and serology analysis was performed on blood samples. PCR was performed on nose and throat swabs. Samples from unexposed controls were collected at a single time point. (**b**) Extent of exposure to COVID-19 index cases in PCR-positive and PCR-negative contacts. Exposure score is a composite of relationship to index score, room sharing score, and index case viral load. Median and quartiles are displayed. p Value of Wilcoxon test is shown. ∗∗p ≤ 0.01. (**c**) Viral load trajectories of PCR-positive contacts. Threshold for PCR-positivity is indicated with a red dashed line.

RNA-seq was performed on longitudinally collected whole blood samples from 48 household contacts of PCR-confirmed COVID-19 index cases. Additionally, 14 unexposed controls were recruited before or early during the COVID-19 pandemic from households comprised entirely of persistently PCR-negative and seronegative individuals. 24 contacts became PCR-positive and seropositive while 24 remained PCR-negative and seronegative throughout follow-up (Figs. 1a, and S1).

All contacts shared communal spaces in a home with a COVID-19 index case. Exposure was further quantified using a score derived from relationship and degree of room sharing with index cases as well as index case viral load (see Methods). PCR-positive contacts had significantly higher exposure scores than PCR-negative contacts, though groups had overlapping score distributions indicating that other factors, in addition to exposure magnitude, influenced risk of PCR-positivity (Fig. 1b). There were no significant differences between PCR-positive and PCR-negative contacts in terms of viral contamination of household surfaces or their index cases’ hands, nor in behavioural changes including hand washing, surface cleaning and room sharing (Table S1). There were no significant differences in age, presence of comorbidities, nor ethnicity between PCR-positive and PCR-negative contacts (Table 1), but the PCR-positive group comprised significantly more males and had higher mean BMI (Table 1).

Initial samples were collected from contacts a median of 3 days after index case symptom onset (IQR 3–4, N = 48 contacts) during ongoing index case viral shedding. Of the 24 PCR-positive contacts, 20 were PCR-positive at enrolment whilst 4 became PCR-positive during the study (Fig. 1c). All PCR-positive contacts seroconverted. There was no evidence of seroconversion in PCR-negative contacts and each PCR-negative contact was seronegative at D0 and D7, excluding the possibility of infection having occurred prior to index case infection (Supplemental Data Sheet). In PCR-positive contacts, to account for the heterogeneity in time of infection relative to enrolment, study timepoints were converted to ‘infection timepoints’ based on when the first PCR-positive swab was collected for a given contact (Table S2). Pre-positive (PP) is used to describe samples collected prior to detectable PCR-positivity and First Positive (FP) describes the timepoint at which the first PCR-positive sample was collected for a given participant. Subsequent timepoints are described relative to FP e.g., FP+7 describes samples collected 7 days after the first PCR-positive sample. For example, study day 0 samples which had a PCR-positive result were assigned to the ‘First Positive’ (FP) infection timepoint and study day 7 samples to ‘First Positive timepoint+7’ (FP+7) infection timepoint. Study day 0 samples which were collected prior to PCR-detectable infection were assigned to the ‘Pre-Positive’ (PP) infection timepoint and their study day 7 sample was assigned to the ‘First Positive’ (FP) infection timepoint. Convalescent samples were collected 28 days post-enrolment.

### Identification of transcriptomic signatures of early SARS-CoV-2 infection

**Fig. 2:**
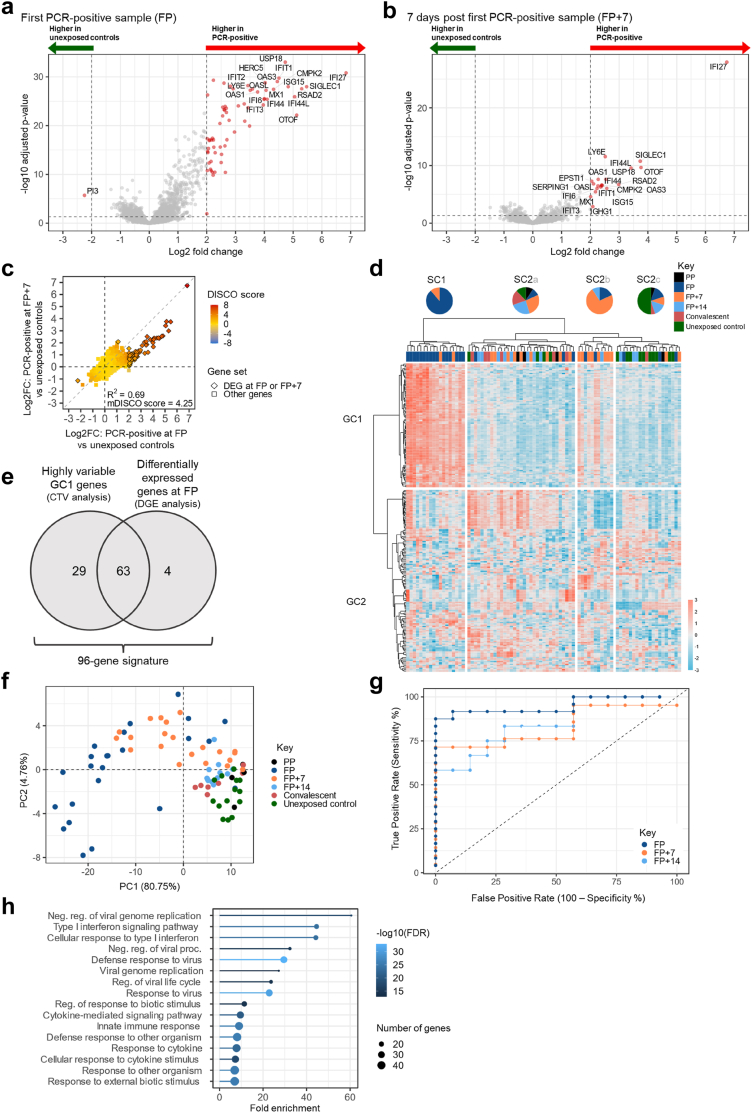
**Identification of a transcriptomic signature of early SARS-CoV-2 infection.** (**a** and **b**) Volcano plots showing differential gene expression between unexposed control (N = 14) and PCR-positive samples at (**a**) FP (N = 24) and (**b**) FP+7 (N = 21). Horizontal dashed line represents the threshold for statistical significance (adjusted p < 0.05). (**c**) Discordance and Concordance (DISCO) plot comparing Log2 fold changes from comparison of samples from unexposed controls to FP samples from PCR-positive contacts to Log2 fold changes from comparison of samples from unexposed controls to FP+7 samples from PCR-positive contacts. Log transformed DISCO scores are indicated by colour. Diamonds outlined in black represent significantly differentially expressed genes (DEGs) from Fig. 2a and b. R^2^ value of Spearman's test and median DISCO score (mDISCO) are displayed. (**d**) Hierarchical clustering heatmap displaying expression of 227 genes with a CTV score >0.0002. Samples from all timepoints from PCR-positive contacts and unexposed controls are included. Red denotes high Z-scored gene expression levels, blue denotes low expression. Study timepoints are annotated using colours indicated in the key and pie charts show the relative contribution of samples from each time point to the number of samples per cluster. Ward clustering linkage and Euclidean distances were used. (**e**) Venn diagram illustrating the overlap between Fig. 2a DEGs and high-variance genes comprising Fig. 2d GC1 identified using CTV analysis. (**f**) Principal component analysis of samples from PCR-positive contacts and unexposed controls using 96-gene signature expression. (**g**) Receiver-operating characteristic (ROC) analysis of median normalised expression of 96-gene signature genes as a predictor of infection status. Median expression in unexposed controls was compared to expression in PCR-positive individuals at the FP, FP+7, FP+14, and convalescent time points yielding AUROC values of 0.95, 0.83, 0.85, and 0.73 respectively. Comparisons where p < 0.05 are displayed. (**h**) Significantly enriched gene ontology terms for the 96-gene signature obtained using ShinyGo. PP = Pre-PCR-positive; FP = Time of First PCR-positive sample; FP+7 = 7 days post FP; FP+14 = 14 days post FP; SC = sample cluster; GC = gene cluster; FDR = false discovery rate.

Differential gene expression analysis was used to compare the transcriptome of samples collected from PCR-positive contacts at the first PCR-positive (FP) timepoint to unexposed healthy control samples (Fig. 2a). 66 genes were upregulated in PCR-positive contacts (log2 fold change > 2, adjusted p < 0.05) and one, *PI3*, was downregulated relative to unexposed controls (log2 fold change < −2, adjusted p < 0.05) (Figs. 2a and S3). At ∼1-week post-infection (FP+7), 20 DEGs were upregulated in PCR-positive contacts relative to unexposed controls (Fig. 2b). Of the 20 DEGs identified at FP+7 only one gene, *IGHG1*, was not also significantly differentially expressed at FP. Gene Ontology (GO) analysis of DEGs showed significant enrichment of pathways related to type I interferon signalling and anti-viral immune response at both FP and FP+7 timepoints (Fig S4a and b).

A Discordance and Concordance (DISCO) analysis was used to assess the degree of concordance between DEGs identified in PCR-positive contacts at FP and those identified at FP+7, both relative to unexposed controls (Fig. 2c; see Methods).^14^^,^^15^ A high degree of concordance was observed with similar genes upregulated at FP and FP+7, though magnitude of upregulation was higher at FP for all but one gene (Fig. 2c).

In parallel to differential gene expression analysis, Contribution to Total Variance (CTV) analysis was used to identify genes that contribute most to the overall variance within the dataset comprised of samples from PCR-positive contacts at all timepoints and unexposed controls (see Methods^13^). This approach does not necessitate *a priori* definition of comparison groups, making it advantageous for capturing genes with heterogeneous expression patterns between individuals and timepoints. A threshold contribution score of 0.0002 was used to define ‘high variance genes’ based on the inflection point of ranked contribution scores for each gene (Figure S5). GO analysis of the resulting 227 high variance genes showed enrichment of key viral defence and immune response pathways, similar to those identified by differential gene expression analysis (Figure S4c). Hierarchical clustering of these genes divided the dataset into two principal gene clusters (GC), GC1 (92 genes) and GC2 (135 genes), and two principal sample clusters (SC), SC1 and SC2 (Fig. 2c). SC1 was comprised entirely of samples collected during acute infection (16/18 FP and 2/18 FP+7) and was characterised by elevated expression of GC1 genes. Three SC2 subclusters were evident—SC2a, SC2b, and SC2c. Interestingly, SC2b primarily consisted of FP+7 samples (8/11 FP+7) and was marked by elevated expression of GC1 genes. The association of GC1 gene upregulation with early SARS-CoV-2 infection was strengthened by the observation that GC1 genes largely overlapped with upregulated genes identified at FP in PCR-positive contacts in differential gene expression analysis (Figure S3).

By combining genes discovered using differential gene expression analysis and those discovered using CTV analysis, we defined a transcriptomic signature of early COVID-19 consisting of 96 genes (Fig. 2e). This signature was made up of 63 genes that were identified in both the differential gene expression analysis and clustered in GC1 in CTV analysis; 4 genes that were uniquely identified using differential gene expression analysis, and 29 genes uniquely identified as CTV analysis CG1 genes (Figs. 2e and S3). High-variance genes identified in CTV analysis that clustered in GC2 were not included in the signature of early COVID-19 as they were not specifically upregulated in early timepoint samples.

We next performed principal component analysis on signature genes (Fig. 2f). Samples collected during acute infection (FP, FP+7) clustered separately from those collected before infection (PP) or after resolution of infection (FP+14, convalescent). PP, FP+14 and convalescent samples clustered with samples from unexposed controls, consistent with this 96-gene signature being a marker of early infection. Receiver-operating characteristic (ROC) analysis using median expression of genes comprising our 96-gene signature showed that expression of signature genes discriminated FP timepoint PCR-positive contacts from unexposed controls, with an area under the ROC (AUROC) of 0.95 (p < 0.0001) (Fig. 2g) but did not discriminate between unexposed controls and convalescent samples from PCR-positive contacts with a significant AUROC. GO analysis of the 96 genes revealed enrichment of multiple antiviral host response pathways, including ‘negative regulation of viral genome replication’ and ‘type I interferon signalling’ (Fig. 2h). Median expression of genes comprising the 96-gene signature correlated positively with viral load at the FP time point, consistent with higher viral load driving increased gene expression (Spearman's R = 0.54, p = 0.008; Figure S6).

### Transcriptomic profile of persistently PCR-negative household contacts of COVID-19 cases

**Fig. 3:**
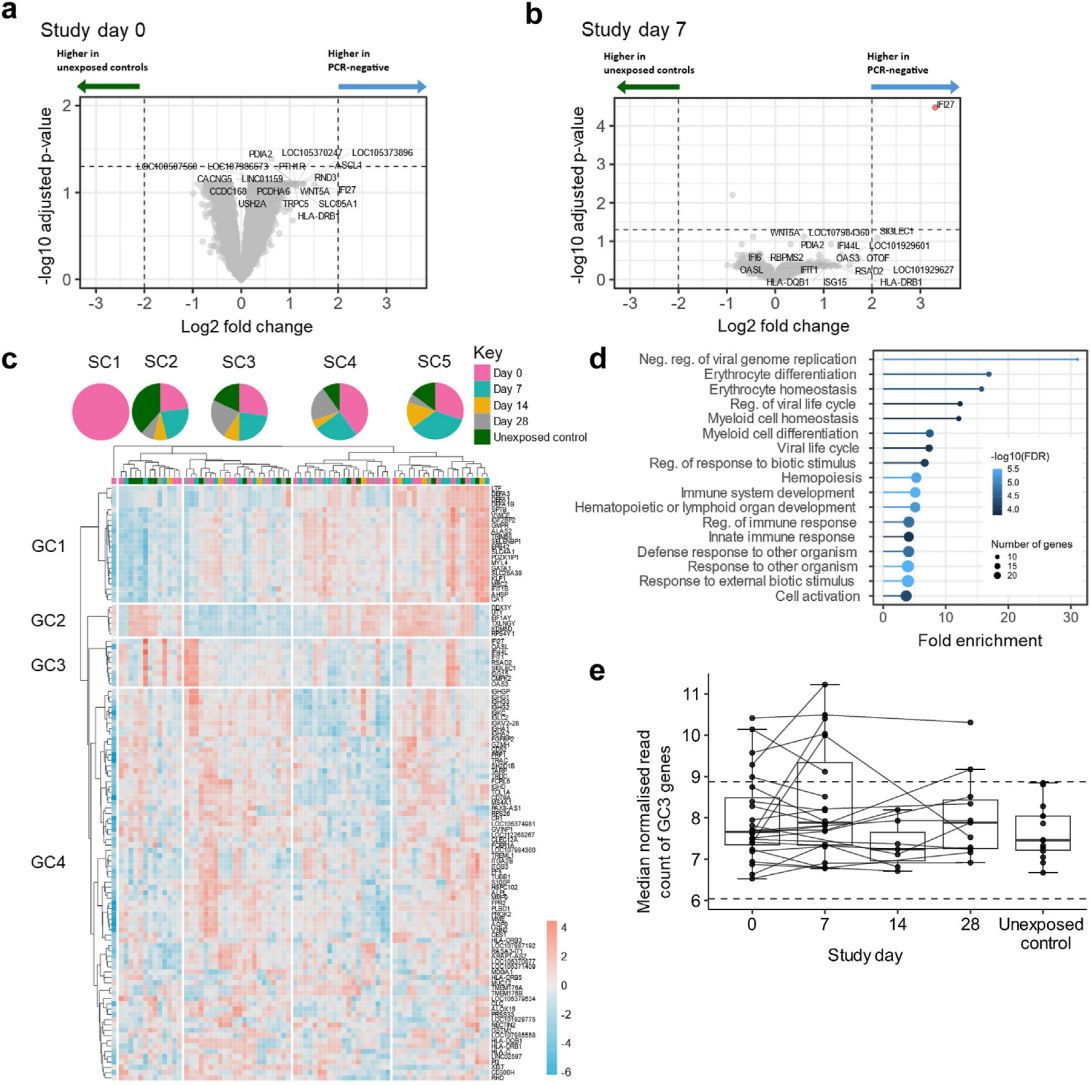
**Transcriptomic profile of persistently PCR-negative COVID-19 household contacts.** (**a** and **b**) Volcano plot showing differential gene expression between unexposed controls (N = 14) and PCR-negative contacts at (**a**) day 0 (N = 24) and (**b**) day 7 (N = 20). Horizontal dashed line represents the threshold for statistical significance (adjusted p < 0.05). (**c**) Hierarchical clustering heatmap of 111 genes with a contribution score >0.0002 in CTV analysis. All samples from PCR-negative contacts and unexposed controls are included. Red denotes high Z-scored gene expression levels, blue denotes low expression. Study timepoints of each sample are annotated using colours indicated in the key and pie charts show the relative contribution of samples from each time point to the total number of samples in each of the labelled clusters. Ward clustering linkage and Euclidean distances were used. (**d**) Significantly enriched gene ontology terms of all 111 CTV analysis-derived high-variance genes from Fig. 3c obtained using ShinyGo. (**e**) Comparison of median normalised read counts of the 9 genes present in GC3 of Fig. 3c from PCR-negative contacts at each study timepoint, and unexposed controls. Median and quartiles are displayed. Two horizontal black dashed lines represent the median GC3 gene expression in unexposed controls ± 2 median absolute deviations. SC = sample cluster; GC = gene cluster; FDR = false discovery rate.

Differential gene expression analysis was performed on data from PCR-negative contacts and unexposed controls to investigate transcriptomic signatures of resistance to infection. No genes were significantly upregulated in persistently PCR-negative contacts at study day 0 compared to unexposed controls (log2 fold change > 2, adjusted p < 0.05; Fig. 3a). At study day 7, *IFI27* was the only significantly upregulated gene in persistently PCR-negative contacts relative to unexposed controls (Fig. 3b). Performing the analysis using a less stringent fold change threshold value of log2 |1| did not result in identification of any additional DEGs, suggesting minimal consistent differences in gene expression profile between highly exposed PCR-negative contacts and unexposed controls.

The discovery of DEGs using differential gene expression analysis relies on similarity in expression profiles within each comparison group and dissimilarity between groups. We hypothesised that the paucity of DEGs between PCR-negative contacts and unexposed controls was caused by high levels of heterogeneity between PCR-negative contacts, and that this variability may be biologically important. To investigate this, CTV analysis was performed to identify genes that contributed most to the total variance across all samples from PCR-negative contacts and unexposed controls. The inclusion of samples collected at multiple timepoints from the same PCR-negative individuals increases the power to identify dynamic transcriptional changes, in addition to variability between individuals. 111 genes had a CTV score >0.0002 and were thus considered high variance genes. Hierarchical clustering based on expression of these genes yielded no obvious clustering of samples by time point nor exposure status (Fig. 3c), however, GO analysis indicated enrichment of several pathways linked to viral infections, with ‘negative regulation of viral genome replication’ being the most enriched pathway (Fig. 3d).

Five of the six genes associated with the ‘negative regulation of viral genome replication’ GO annotation appeared in GC3 and all 9 GC3 genes also appeared in the 96-gene signature of early infection (Figs. 3c and S3), suggesting that GC3 comprised genes associated with early SARS-CoV-2 infection. Moreover, we noted that higher GC3 gene expression tended to occur in early timepoint samples from PCR-negative contacts and not in unexposed controls (Fig. 3c). The median normalised read count of GC3 genes in PCR-negative contacts was compared to unexposed controls to investigate whether this cluster of genes was upregulated in those who resist infection. We observed no statistically significant difference in GC3 gene expression between unexposed controls and persistently PCR-negative contacts at any timepoint using multiple Mann Whitney U tests (Fig. 3e). However, GC3 expression was elevated at D0 and D7 in 5 and 6 individuals respectively relative to the median + (2 × median absolute deviations) of unexposed controls, with 4 individuals having elevated GC3 expression at both D0 and D7. This suggests that activation of innate antiviral pathways during SARS-CoV-2 exposure occurs in a proportion of PCR-negative contacts.

### An early SARS-CoV-2 infection signature identifies immune resistance to infection in a proportion of persistently PCR-negative COVID-19 household contacts

**Fig. 4:**
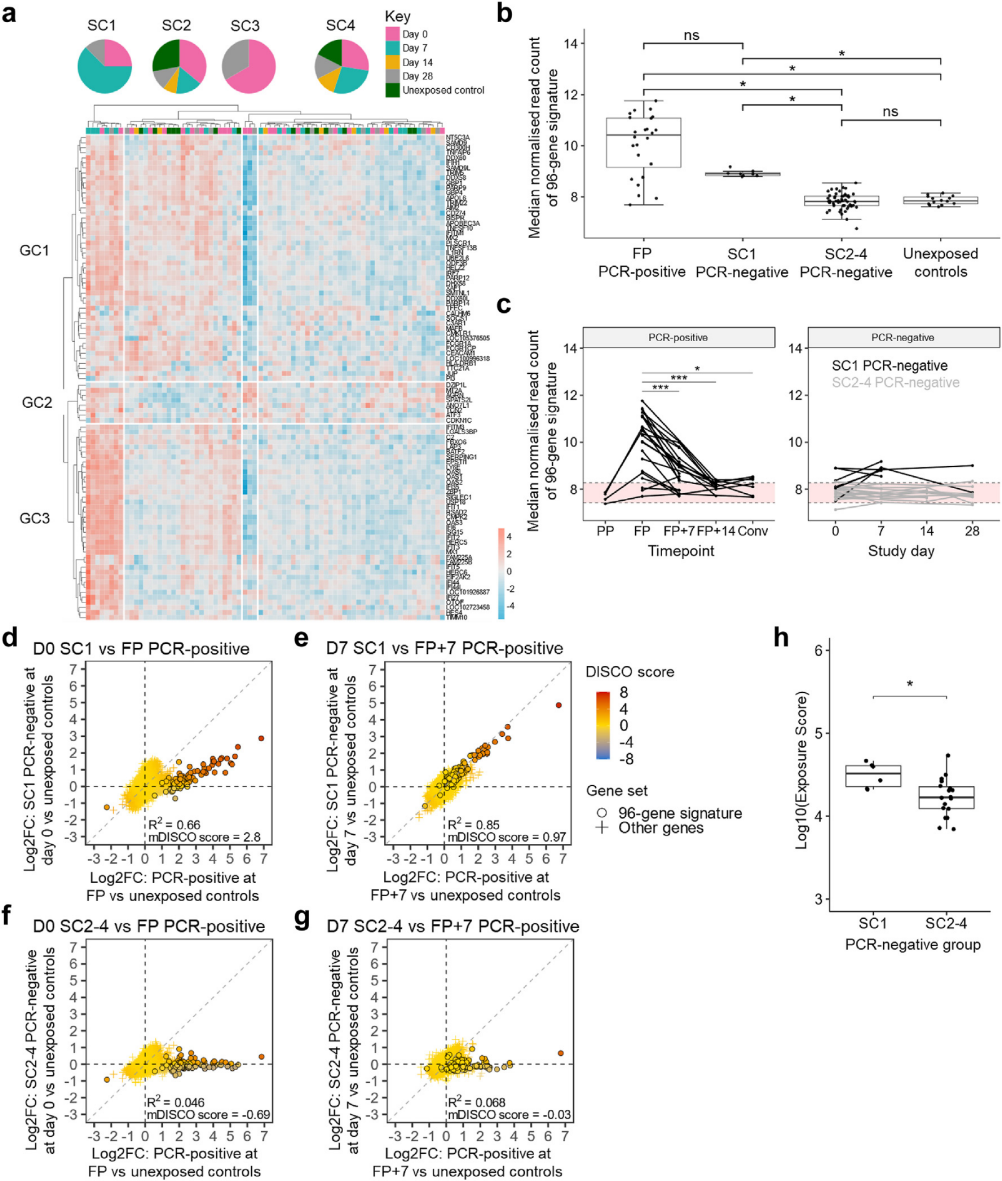
**An early SARS-CoV-2 infection signature identifies immune resistance to sustained infection in a subgroup of persistently PCR-negative COVID-19 household contacts.** (**a**) Hierarchical clustering of 96-gene signature in PCR-negative contacts and unexposed controls. Red denotes high Z-scored gene expression levels; blue denotes low expression. Study timepoints are annotated using colours indicated in the key and pie charts show the relative contribution of samples from each time point to the number of samples per cluster. Ward clustering linkage and Euclidean distances were used. (**b**) Comparison of median normalised read counts of 96-gene signature between unexposed samples, PCR-positive contact FP samples and samples from PCR-negative contacts stratified by Fig. 4a sample cluster. Median 96-gene signature expression was compared using Dunn's Test with Bonferroni correction. ∗p ≤ 0.05. (**c**) Median normalised read counts of 96-gene signature genes over time. PCR-positive contacts and SC1 PCR-negative contacts from Fig. 4a are shown in black. Other PCR-negative contacts are shown in grey. Red bands represent median normalised 96-gene signature gene count in unexposed controls ±2 median absolute deviations. Mixed effects analysis with Tukey's multiple comparisons is displayed. ∗p ≤ 0.05, ∗∗∗p ≤ 0.001. **(d–g)** Discordance and Concordance (DISCO) plots comparing fold change in gene expression relative to unexposed controls samples between: (**d**) SC1 PCR-negative contact day 0 samples and PCR-positive contact FP samples; (**e**) SC1 PCR-negative contact D7 samples and PCR-positive contact FP+7 samples; (**f**) SC2-4 PCR-negative contact day 0 samples PCR-positive contact FP samples; and (**g**) SC2-4 PCR-negative contact day 7 samples and PCR-positive contact FP+7 samples. Log transformed DISCO scores are indicated by colour. Genes comprising the 96-gene signature are represented by black outlined circles. Other genes are represented by crosses. R^2^ value of Spearman's tests and median DISCO scores (mDISCO) for 96-gene signature genes are displayed. (**h**) Comparison of COVID-19 index case exposure score between PCR-negative contacts with samples in Fig. 4a SC1 and those without SC1 samples using Wilcoxon test ∗p ≤ 0.05. Box plots show median and quartiles. PP = Pre-PCR-positive; FP = Time of First PCR-positive sample; FP+7 = 7 days post FP; FP+14 = 14 days post FP; Conv = Convalescent; D0 = Study day 0; D7 = Study day 7; SC = sample cluster; GC = gene cluster; ns = non-significant.

To test whether responses associated with early replicative infection may be associated with resistance to detectable infection, hierarchical clustering of expression of the 96-gene signature presented in Figs. 2e and S3 was performed on samples from persistently PCR-negative contacts and unexposed controls. Samples segregated into four main clusters (Fig. 4a). Sample cluster 1 (SC1) was characterised by high levels of expression of early infection signature genes with GC1 and GC3 genes particularly upregulated. SC2-4 had variable levels of GC1 expression and generally lower levels of GC3 gene expression. SC1 consisted of 8 samples from 6 persistently PCR-negative contacts; 7 of these 8 samples were collected on study days 0 and 7, during the period of acute SARS-CoV-2 exposure.

96-gene signature expression in SC1 PCR-negative samples was significantly higher than in both SC2-4 PCR-negative samples and unexposed control samples (Fig. 4b). Conversely, 96-gene signature expression was not significantly different between SC1 PCR-negative samples and FP samples from PCR-positive contacts (Fig. 4b), consistent with antiviral immune activation in both groups. 96-gene signature expression was elevated in 22/24 PCR-positive contacts relative to a threshold derived from median signature gene expression in unexposed control + 2 × median absolute deviations (Fig. 4c). This threshold was also exceeded in 8/24 persistently PCR-negative contacts (Fig. 4c). Of these, 6 individuals were represented in SC1 in Fig. 4a. The median normalised read count was significantly different between contacts with SC1 PCR-negative samples and healthy controls only at the day 7 timepoint, suggesting dynamic expression of the 96-gene signature in these contacts (Multiple Mann–Whitney U-test with Benjamini, Krieger, and Yekutieli 5% FDR q < 0.01).

DISCO analysis revealed high concordance in gene expression profile between SC1 PCR-negative contacts and PCR-positive contacts, but poor concordance between SC2-4 PCR-negative contacts and PCR-positive contacts (Fig. 4d–g). Genes comprising the 96-gene signature were particularly concordant, exemplified by a strong positive correlation between 96-gene signature expression when comparing D7 samples from SC1 PCR-negative contacts to FP+7 samples from PCR-positive contacts (R^2^ = 0.85) (Fig. 4e). Differential gene expression analysis comparing SC1 PCR-negative contacts to SC2-4 PCR-negative contacts yielded no upregulated genes in the SC1 group other than 96-gene signature genes (Figure S7).

No significant differences in demographic characteristics were evident between SC1 PCR-negative contacts and other PCR-negative contacts (Table 2). However, SC1 PCR-negative contacts had significantly higher exposure scores compared to SC2-4 PCR-negative contacts (Fig. 4h). This effect was driven by SC1 PCR-negative contacts having a closer relationship to their respective index cases and higher room share scores, rather than differences in index case viral load (Table S3).

**Table 2:** Demographic data of PCR-negative contacts using sample clusters defined in Fig. 4a.

	Total (N = 24)	SC1 PCR-negative contacts (N = 6)	Other PCR-negative contacts (N = 18)	p-Value
**Sex**				0.967
Female	17 (70.8%)	4 (66.7%)	13 (72.2%)	
Male	7 (29.2%)	2 (33.3%)	5 (27.8%)	
**Age (years)**				0.961
<18	2 (8.3%)	0 (0%)	2 (11.1%)	
18–39	9 (37.5%)	2 (33.3%)	7 (38.9%)	
40–59	12 (50.0%)	4 (66.7%)	8 (44.4%)	
≥60	1 (4.2%)	0 (0%)	1 (5.6%)	
**BMI**^a^				0.674
Normal weight	15 (62.5%)	3 (50.0%)	12 (66.7%)	
Overweight	6 (25.0%)	3 (50.0%)	3 (16.7%)	
Obese	1 (4.2%)	0 (0%)	1 (5.6%)	
Missing	2 (8.3%)	0 (0%)	2 (11.1%)	

p Values of χ^2^ tests used to compare groups are displayed.

a BMI thresholds are as follows: normal weight ≥18.5 & <25; overweight ≥25 & <30; and obese ≥30. BMI is excluded for individuals under the age of 18.

### The early SARS-CoV-2 infection signature identifies immune resistance to infection in two additional, independent cohorts

**Fig. 5:**
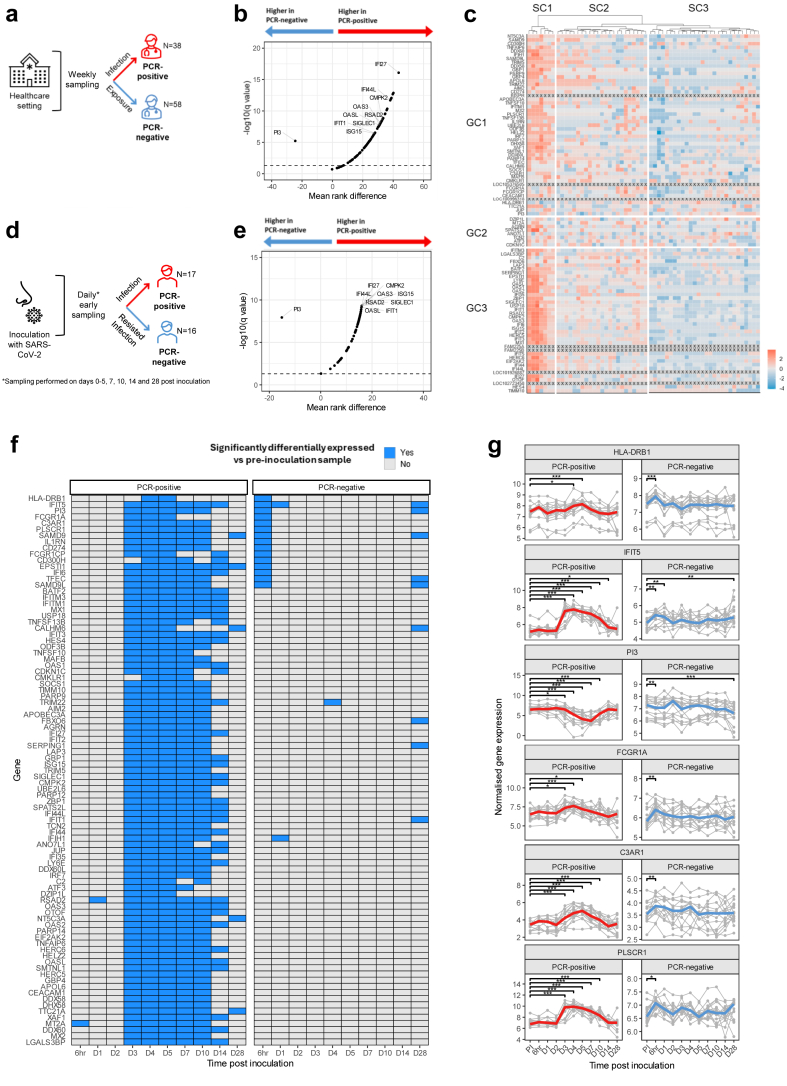
**Early SARS-CoV-2 infection signature identifies cases of immune resistance to sustained infection in a separate, independent validation cohort.** (**a**) Diagrammatic representation of COVIDsortium study design. RNA-seq was performed on whole blood samples from N = 38 PCR-positive and N = 58 PCR-negative healthcare workers (HCWs) with presumed high magnitude exposure to SARS-CoV-2. (**b**) Volcano plot representing results of Multiple Mann Whitney U tests comparing expression levels of 89 genes from the 96-gene signature between PCR-positive and PCR-negative HCWs. 7 of the 96-gene signature genes were not annotated in this dataset. Horizontal dashed line represents the threshold for statistical significance (FDR < 0.05 after 2-stage multiple hypothesis correction (Benjamini, Krieger, and Yekutieli)). 9 Fig. 3c GC3 genes PI3 are labelled. (**c**) Hierarchical clustering of the truncated 96-gene signature in PCR-negative HCWs. Genes are displayed in the order generated by hierarchical clustering displayed in Fig. 4c. Red denotes high Z-scored gene expression levels; blue denotes low expression. Black crosses represent the 7 genes from the 96 gene signature that were not present in the COVIDsortium dataset. Ward clustering linkage and Euclidean distances were used. SC = sample cluster; GC = gene cluster. (**d**) Diagrammatic representation of CHIM study design. RNA-seq was performed on longitudinal whole blood samples from individuals intranasally inoculated with SARS-CoV-2. N = 17 were seronegative at the time of inoculation and became PCR-positive and seroconverted. N = 16 were seronegative at the time of inoculation, remained PCR-negative at serial time points and did not seroconvert. (**e**) Volcano plot representing results of Multiple Mann Whitney U tests comparing expression levels of 89 genes from the 96-gene signature between PCR-positive and PCR-negative CHIM participants. 7 of the 96-gene signature genes were not annotated in this dataset. Horizontal dashed line represents the threshold for statistical significance (FDR < 0.05 after 2-stage multiple hypothesis correction (Benjamini, Krieger, and Yekutieli)). 9 Fig. 3c GC3 genes and PI3 are labelled. (**f**) Tile plot displaying the results of multiple paired Mann Whitney U-tests comparing expression of each of the indicated genes pre-inoculation to post-inoculation at each of the indicated time points. Analysis was conducted independently for PCR-positive and PCR-negative participants. Multiple comparisons are corrected for using the Benjamini–Hochberg method. Correction was applied for both comparison of multiple post-inoculation time points and comparisons of multiple genes. Blue tiles represent instances where significant differences (p < 0.05) were observed post-correction. (**g**) Line graphs displaying the dynamics of expression of genes that were most differentially expressed in PCR-negative contacts between pre-inoculation and 6 hr post-inoculation samples. Grey lines represent expression of genes in individual participants. Bold coloured lines indicate the median gene expression. Red lines = PCR-positive participants; blue lines = PCR-negative participants. Asterisks indicate significant differences in expression compared to pre-inoculation expression (Multiple Mann–Whitney U-test with Benjamini–Hochberg correction. ∗p < 0.05, ∗∗p < 0.01, ∗∗∗p < 0.001.) PI = Pre-inoculation, 6 hr = 6 hours post-inoculation.

We next investigated expression of our 96-gene signature in published RNA-seq data from two additional cohorts; the COVIDsortium cohort of highly SARS-CoV-2-exposed healthcare workers (HCWs); and a SARS-CoV-2 controlled human infection model (CHIM) study. The former comprises data from 96 HCWs of whom 38 became infected (ArrayExpress: E-MTAB-10022) and the latter comprises data from 36 healthy individuals intranasally inoculated with 10 TCID_50_ wild type SARS-CoV-2, 18 of whom became infected (ArrayExpress: E-MTAB-12993) (Fig. 5a–d).^18^^,^^19^ Both datasets were first used to validate our 96-gene signature as a marker of sustained, replicative COVID-19 infection. Read counts were available for 89 of 96 (93%) of the genes comprising our early infection gene signature in both datasets. In the COVIDsortium dataset, 81 of these 89 genes (91%) were expressed at significantly higher levels in PCR-positive compared to PCR-negative HCWs (Fig. 5b). In the CHIM dataset, 88 of these 89 (99%) genes were significantly upregulated in PCR-positive cases compared to PCR-negative cases at D7 post-inoculation, when peak nasal viral load occurred (Fig. 5e).^19^ In both COVIDsortium and D7 CHIM data, *PI3* was significantly upregulated in PCR-negative cases relative to PCR-positive cases, as observed in our household cohort (Fig. 5b and e). These findings validate the generalisability of our early infection signature derived from household contacts.

We next investigated whether SARS-CoV-2-exposed persistently PCR-negative HCWs also expressed elevated expression of genes associated with early infection, similar to the subset of PCR-negative household contacts represented in SC1 in Fig. 4a. Hierarchical clustering of 58 samples from PCR-negative HCWs based on expression of genes in the truncated 89-gene signature revealed a cluster of 7 individuals (SC1) that expressed high levels of these genes (Fig. 5c). Both these 7 HCWs and household contacts of COVID-19 cases represented in cluster SC1 in Fig. 4a share a phenotype of persistent PCR-negativity despite SARS-CoV-2 exposure and both upregulate innate immune pathway genes following SARS-CoV-2 exposure, suggesting recent effective immunological containment of infection.

To further test the hypothesis that signature gene expression is associated with resistance to infection and to investigate expression dynamics in those who resist infection, we used paired multiple Mann–Whitney U-Test analyses to investigate changes in signature gene expression pre- and post-SARS-CoV-2 inoculation in 16 persistently seronegative CHIM participants. In these individuals, who were definitively SARS-CoV-2-exposed by direct intranasal inoculation, we observed significant increases in expression of 14 signature genes 6 hr post-inoculation (Fig. 5f). We also found contemporaneous significant downregulation of *PI3*. Interestingly, this modulation of expression was very transient, with only 2 genes being significantly upregulated at D1, and 0 genes at D2 and D3.

We next compared the pre- and post-inoculation dynamics of gene expression in PCR-negative and PCR-positive participants (Fig. 5g). In contrast to the PCR-negative participants, the PCR-positive participants exhibited no upregulation of signature genes at 6 hr post-inoculation; 1 of the 89 signature genes, *MT2A*, was significantly downregulated at 6 hr post-inoculation (Figure S8). Indeed, signature genes were not significantly upregulated until 3 days post-inoculation in PCR-positive participants, whereupon all signature genes became durably upregulated. As in our household contact cohort, the magnitude of signature gene expression in PCR-positive individuals was considerably higher than in early time-point samples from PCR-negative individuals.

## Discussion

We present an IFN-associated 96-gene blood transcriptional signature of recent SARS-CoV-2 exposure which was strongly upregulated in recently infected COVID-19 contacts across three independent cohorts from diverse settings, with the temporal dynamics of the response closely following the *in vivo* SARS-CoV-2 viral load. The signature was also dynamically expressed in one quarter of SARS-CoV-2-exposed persistently PCR-negative seronegative household contacts, though at lower magnitude. Hence, in addition to being a reproducible, dynamic biomarker of recent SARS-CoV-2 infection, this transcriptomic signature also identifies individuals with recent high-level SARS-CoV-2 exposure who do not develop overt, sustained infection. Additionally, signature genes were significantly upregulated in uninfected CHIM participants. The presence of this dynamic signature in CHIM participants who do not become infected is important because, unlike the household contacts and HCWs who were deemed to have been exposed by virtue of their close household and nosocomial contact respectively, the CHIM participants were definitively exposed by direct intranasal inoculation of virus.

Whilst gene signature expression in PCR-negative contacts from our household contact cohort represents a marker of recent exposure, it does not constitute a correlate of protective immunity. This is because similar genes were upregulated in both PCR-positive and PCR-negative contacts, albeit at different magnitudes. Conversely, in the CHIM study, while signature genes were upregulated in both PCR-positive and PCR-negative contacts, there was a stark difference in the timing of gene expression. We observed significant, ultra-early (6 hr) transient increases in expression of signature genes post-inoculation in PCR-negative volunteers while in PCR-positive volunteers significant upregulation did not occur until 3 days post-inoculation. Therefore, in this context, very early transient upregulation of signature genes post-SARS-CoV-2 inoculation distinguishes persistently PCR-negative volunteers, identifying a potential correlate of protection against sustained infection.

Differences in the nature of SARS-CoV-2 exposure between household contacts and CHIM volunteers likely impact the dynamics of expression of our gene signature. In CHIM studies, a defined titre of a single strain of laboratory-propagated virus is administered at a single time point in a single anatomical compartment, the nares. In contrast, household contacts are exposed to unquantified numbers of virions of heterogenous viability and replicative potential through serial exposure events occurring across the infectious window of the index case.^7^ Moreover, exposure likely occurs more generally across the upper respiratory tract, including exposure of the nares and oropharynx to virions in inhaled aerosol, and through other mucosal routes, i.e., exposure of mucous membranes including the conjunctivae to virions transferred by hand from contaminated surfaces (fomite spread).^1^ We postulate that these differences in dose, route, timing and duration of SARS-CoV-2 exposure explain the more protracted signature expression observed in PCR-negative household contacts compared to remarkably short-lived expression in PCR-negative CHIM participants.

**Fig. 6:**
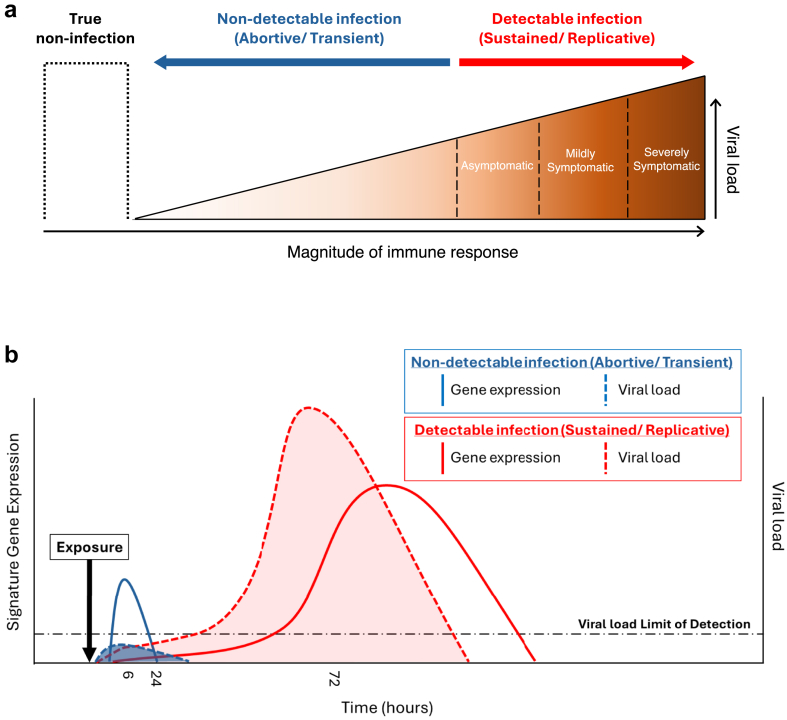
**Hypothesised model of outcomes to SARS-CoV-2 exposure.** (**a**) Schematic representation of a model of the spectrum of outcomes to SARS-CoV-2 exposure. Exposure and infection exist on a spectrum in which a proportion of contacts resist establishment of sustained replicative infection. In this model, increasing magnitude of viral exposure drives increased innate immune pathway activation. A proportion of contacts avoid viral entry into cells lining the upper respiratory tract, hence experience ‘true non-infection’ and have no measurable innate immune response to exposure. Other contacts experience abortive infection in which virus is rapidly eliminated and is undetectable by conventional PCR, or transient infection in which virus is transiently detectable by conventional PCR but is optimally controlled and does not induce seroconversion. We hypothesise that in these cases, the immune response optimally limits viral replication. This is detectable in the periphery at a magnitude commensurate with the quantity of virus a contact was exposed to. In contacts who fail to optimally control early infection, detectable, sustained replicative infection occurs. In this case intra-host viral replication determines the magnitude of the innate immune response i.e., magnitude of innate immune response correlates positively with peak viral load. We hypothesise that similar immune pathways are induced in those who resist replicative infection and those who become infected, though at different magnitudes. Further, we hypothesise that it is the speed and magnitude of the early, local immune responses that determine whether an individual succumbs to sustained replicative infection. ∗Detectable infection is defined using conventional PCR and serology methods. (**b**) Schematic representation of the dynamics of viral load and signature gene expression in CHIM participants over time, comparing those who resist detectable infection (blue) with those who succumb to detectable infection (red). Solid lines represent signature gene expression. Dashed lines represent viral load. Horizontal dashed line represents limit of detection for viral load measurement. A rapid increase in expression of signature genes in those with non-detectable infection (blue solid line) is sufficient to limit viral load such that it is undetectable by PCR (blue dashed line). Conversely, slower induction of signature genes in those with detectable infection fails to control early viral proliferation which continues exponentially, driving further expression of signature genes.

Based on our observations we propose a model in which the strength of the innate immune response, measured in our study by level of expression of our IFN-associated 96-gene signature, increases in response to the quantity of virus interacting with the host immune system in the upper respiratory mucosa. This is supported by our observation of a positive correlation between early viral load and 96-gene signature expression intensity. We propose that this phenomenon extends beyond PCR-positive individuals to highly exposed PCR-negative contacts, explaining the observation that persistently PCR-negative household contacts with the highest level of signature expression (SC1 samples from Fig. 4a) had the highest magnitude SARS-CoV-2 exposure. The spectrum of outcomes following SARS-CoV-2 exposure ranges from true non-infection as a result of either avoiding contact between virions and epithelial cells, to abortive (i.e., undetectable) or transient (i.e., transiently detectable) infection, to detectable, sustained, replicative infection. We propose that the magnitude of IFN-associated immune gene expression mirrors this spectrum of *in vivo* respiratory mucosal viral load (Fig. 6a).

Our findings from CHIM data support our model, suggesting that in those who resist infection, a very early IFN-associated response within hours of viral exposure is sufficient to limit and abrogate early viral replication when viral load is still low, such that infection is not established and cannot be detected by PCR. In those who develop sustained infection, however, these conserved innate immune responses are not activated until 3–4 days later by which time unchecked exponential viral proliferation results in established infection with high viral load that subsequntly drives higher-magnitude immune responses through the course of infection (Fig. 6b).

Whether the genes comprising our 96-gene signature are themselves involved in protecting from overt replicative infection in persistently PCR-negative cases is an important question. Although this cannot be directly tested in observational studies, the composition of the signature suggests potential for constituent genes to mediate viral containment. Most signature genes are constituents of antiviral pathways, in particular IFN signalling which is induced upon SARS-CoV-2 binding to RIG-I-Like Receptors and Toll-Like Receptors and has been shown to limit viral replication and associate with less severe outcomes in hospitalised COVID-19 cases.^20^, ^21^, ^22^, ^23^, ^24^, ^25^, ^26^

Of the 14 genes significantly upregulated at 6 hr post-inoculation in PCR-negative but not PCR-positive CHIM participants (*HLA-DRB1*, *IFIT5*, *FCGR1A*, *C3AR1*, *PLSCR1*, *SAMD9*, *IL1RN*, *CD274*, *FCGR1CP*, *CD300H*, *EPSTI1*, *IFI6*, *TFEC*, *SAMD9L*), 8 are canonical ISGs and elevated expression of three, *IFIT5*, *IFI6*, and *PLSCR1*, has been shown to restrict SARS-CoV-2 growth in *in vitro* overexpression or loss of function studies.^27^, ^28^, ^29^, ^30^ Each of these negative regulators of SARS-CoV-2 growth acts at a different stage of viral replication; IFIT5 interferes with viral replication through RNA binding,^28^ IFI6 acts at the endoplasmic reticulum potentially affecting formation of replication organelles,^31^ and PLSCR1 blocks late-stage SARS-CoV-2 entry, a role that is conserved across host species and is SARS-CoV-2 variant-agnostic.^29^ CRISPR-based knockdown studies to identify Flavivirus and Influenza virus restriction factors have also shown IFI6 and PLSCR1 to be key inhibitors of replication of these viruses respectively.^31^^,^^32^ These observations support a conserved protective role for these and potentially other genes among the 14 that are rapidly, transiently upregulated in CHIM participants who resist infection.

Interestingly, one gene, *PI3*, which was downregulated in PCR-positive household contacts and HCWs, was significantly, transiently downregulated 6 hr post-inoculation in PCR-negative but not PCR-positive CHIM participants in whom it was not downregulated until 3 days post-inoculation. *PI3* downregulation thus followed the same kinetic as the 14 transiently upregulated protection-associated genes. The role of *PI3* in SARS-CoV-2 control is unknown, though its protein product, Elafin, is secreted in response to microbial pathogens, controlling neutrophil-mediated extracellular matrix degradation by inhibiting neutrophil elastase, resulting in prevention of excess tissue damage.^33^^,^^34^ Whether and how early transient *PI3* downregulation might contribute to protection against infection remains to be determined.

Pre-existing, cross-reactive T cells have been associated with resistance to SARS-CoV-2 infection despite exposure.^35^^,^^36^ Swadling et al.^36^ showed that SARS-CoV-2-naïve HCWs who resisted infection frequently possessed SARS-CoV-2 replication transcription complex (RTC)-specific T cells, implicating these pre-existing, cross-reactive T cells as mediators of resistance to infection. Interestingly, HCWs with a strong anti-RTC T cell response had significantly higher levels of *IFI27* expression. Mechanistically, these observations could be explained by arrest of SARS-CoV-2 replication in respiratory epithelial cells at the stage of RNA-dependent RNA polymerase negative sense RNA synthesis by cell intrinsic mechanisms such as RIG-I binding to viral positive sense template RNA.^37^ This process would not, however, result in inhibition of transcription of RTC components which could still be processed and presented to cognate T cells driving the post-exposure RTC-specific T cell expansion observed by Swadling et al.^36^ Thus, in individuals with pre-existing, cross-reactive T cells, both the T cells and early innate immune responses likely mediate protection against infection, though their relative contributions are unknown.

Although there is clearly potential for innate and cross-reactive adaptive arms of the immune system to cooperate to mediate resistance to sustained infection, it is also possible that innate immune responses alone, including for example pathways associated with our 96-gene signature, are sufficient. We observed no evidence of SARS-CoV-2 antigen-specific adaptive immunity associating with resistance to infection at the pathway level in any of the GO-based analyses performed, consistent with scRNAseq-seq data from CHIM studies showing no evidence for adaptive immune cell involvement in protection against infection.^38^

Several limitations must be considered when interpreting our findings. We measured transcriptomic events in the periphery, yet the respiratory mucosa is the initial site of host-pathogen interaction. Rather than mediating resistance to infection, signatures observed in blood may be a marker of a protective local response at the mucosal site of exposure. Future research involving longitudinal analysis of mucosal samples from virus-exposed individuals and animal models will be required to fully elucidate the biological mechanisms underlying resistance to infection. Timing of sample collection in our household study is also an important limitation of our study. Heterogeneity in time of infection relative to enrolment in PCR-positive contacts could have affected the composition of the 96-gene signature we derived, as transiently expressed genes would be less likely to be detected. Additionally, since samples in our household cohort were not collected daily it is impossible to exclude the possibility that some PCR-negative contacts may have been transiently weakly PCR-positive at timepoints that we did not sample e.g., days 2–3 or 5–6 post-enrolment. However, whether some of these contacts had undetected transient or truly undetectable abortive infections does not significantly impact our findings. In both cases, the infection is asymptomatic, not sustained, does not trigger seroconversion, and is very likely non-infectious. Importantly, the contacts have, in both scenarios, mounted a successful early host response that eliminates virus before establishment of sustained, replicative infection.

We were unable to exclude infection with pathogens other than SARS-CoV-2. Though infection with other pathogens is a potential explanation for the 96-gene signature expression observed in SC1 PCR-negative contacts, it is unlikely because these individuals were asymptomatic, had no known exposure to other pathogens, and a low pre-enrolment probability of exposure due to strict limitations placed on social contact during the period of sample collection.^39^ Whilst sex differences in baseline and SARS-CoV-2 infection-induced ISG expression have been demonstrated, we observed no statistically significant differences in 96-gene signature intensity between male and female household contacts at any time point regardless of whether contacts were PCR-positive or -negative. However, we were unable to conclusively exclude an effect of biological sex on signature expression due to insufficient statistical power as a result of relatively small sample size (data not shown).^40^ Finally, data relating to the timing, frequency, and nature of exposure events in the HCW cohort used to validate our 96-gene signature were limited, precluding assessment of the dynamic relationship between SARS-CoV-2 exposure and signature gene expression in these individuals.

Our findings exemplify the power of triangulation between real-world community studies of natural virus exposure and highly controlled experimental human infection models. We exploited the unique advantages of these very distinct study designs to identify a robust, reproducible gene signature of recent SARS-CoV-2 infection that is also present in highly-exposed, uninfected individuals. We further showed that a subset of genes comprising our transcriptomic signature are transiently upregulated within hours of viral inoculation in CHIM participants who remain PCR-negative, but not in those who become infected, supporting the conclusion that very early expression of these genes correlates with, and may contribute to, protection from infection. Given their highly conserved roles, these protection-associated IFN-related innate pathways are likely relevant to a broad range of SARS-CoV-2 variants and, potentially, other respiratory viruses. This paves the way for translational studies to test the prophylactic and therapeutic potential of early interventions to stimulate these innate antiviral pathways in contacts recently exposed to SARS-CoV-2 variants and other respiratory viruses.

## Contributors

INSTINCT study group investigators contributed to participant recruitment and enrolment, collection and biobanking of biological samples, and management, administrative, and logistical aspects of the INSTINCT study.

Conceptualisation: JF, AL, RD, KM, EC, RK, TDP, SH.

Software: KM, RD, EC.

Validation: JF, KM, EC.

Formal analysis: JF, KM, EC, RD.

Investigation: JF, KM, EC, RD, SN, RK, SH, AK, ND, MTW, JJ, LW, SB, TDP, CL, RV, AB, EP, CR, MM, RT, GT, AL.

Data curation: JF, KM, EC, RD, SN, RK, SH, AK, ND, MTW, JJ, LW, SB, TDP, CL, RV, AB, EP, CR, MM, RT, GT, AL.

Writing – original draft: JF, EC, KM, RD.

Writing – review & editing: JF, EC, KM, RD, SH, SN, AK, JJ, JST, RST, AL.

Visualization: KM, EC, JF, RD.

Supervision: JF, GT, RT, MM, AL.

Project administration: AL.

Funding acquisition: AL, JF, SH, RT, MM.

The following authors accessed and verified that data underlying data presented in this manuscript: JF, KM, EC, RD.

All authors have read and approved the final version of this manuscript.

## Data sharing statement

Anonymised, de-identified versions of datasets presented in this manuscript are available in the Supplemental Data Sheet. Additional data is available upon request to the corresponding author. INSTINCT study documents including study protocol, participant case record form (CRF), informed consent form and participant information sheet (PIS) are available upon request. Code related to the Contribution to Total Variance (CTV) analysis pipeline presented and related data can be found on the GitHub repository (https://github.com/rderelle/SCBD). RNA-seq data generated from this paper have been deposited to the European Genome-Phenome Archive (EGA), Dataset ID: EGAD50000000684 (https://ega-archive.org/datasets/EGAD50000000684).

## Declaration of interests

RT and MM report patent pending (Patent Application No. 2011047.4 for “SARS-CoV-2 antibody detection assay”). All other authors declare no competing interests.

## Appendix ASupplementary data

Figures S1–S8 Figure S1: **Cohort inclusion criteria.** Flowchart detailing the derivation of the cohort used in subsequent analyses from total INSTINCT study recruitment. ∗PCR-positive contact defined as having a PCR-positive result at 1 or more study timepoints. †PCR-negative contact defined as being persistently PCR-negative and seronegative at all timepoints with available data. Figure S2: **Graphical representation of analytical pipeline.** Type of analysis and groups of samples analysed for each figure are shown in separate, coloured boxes. FP = Time of First PCR-positive sample; FP+7 = 7 days post FP; UC = unexposed control; GC = gene cluster; SC = Sample cluster; HCW = Healthcare worker; CTV = Contribution to total variance; DGE = Differential gene expression; GO = Gene Ontology. Figure S3: **Visualisation of overlap of gene lists.** Heatmap indicating analyses in which genes comprising the 96-gene signature indicated in Fig. 3c were identified. Columns represent specific analyses presented in figures in the manuscript. Blue indicates where genes were identified, orange indicates where genes were not identified. FP = Time of First PCR-positive sample; FP+7 = 7 days post FP; DEG = differentially expressed genes; CTV = contribution to total variance; GC = gene cluster; HCW = healthcare worker. Figure S4: **Gene Ontology (GO) analysis of gene lists derived from analyses of PCR-positive contacts vs unexposed controls.** (**a**) Enriched GO terms for the 67 DEGs between FP samples from PCR-positive contacts and unexposed control samples depicted in Fig. 2a. (**b**) Enriched GO terms for the 20 DEGs between FP+7 samples from PCR-positive contacts and unexposed control samples depicted in Fig. 2b. (**c**) Enriched GO terms for the 227 high-variance genes identified from CTV analysis of all samples from PCR-positive contacts and unexposed controls depicted in Fig. 2d. All graphs produced using ShinyGo. FP = Time of First PCR-positive sample; FP+7 = 7 days post FP; DEG = differentially expressed genes; CTV = contribution to total variance; FDR = false discovery rate. Figure S5: **Contribution to total variance (CTV) scores for all genes from CTV analysis of samples from PCR-positive contacts and unexposed controls.** Genes were ranked according to their CTV score. A threshold of 0.0002, indicated by a red dashed line, was used to determine high-variance genes based on infection point of the plot. Genes with a score >0.0002 were used in downstream analyses. Figure S6: **96-gene signature correlates with FP viral load.** Spearman’s correlation of median gene expression of all genes comprising the 96-gene signature at FP with log10 viral load at FP in N = 24 PCR-positive contacts. Spearman’s R and p value are displayed. Grey shaded region indicates 95% confidence interval. FP = Time of First PCR-positive sample. Figure S7: **Differential gene expression analysis of S1 and S2-4 PCR-negative contacts.** Volcano plot showing differential gene expression between SC1 PCR-negative contacts and SC2-4 PCR-negative contacts at (**a**) day 0 and (**b**) day 7. Adjusted p value < 0.05 was used as the significance threshold. Figure S8: **Line graphs showing dynamics of gene expression in CHIM participants.** Line graphs display expression of all genes that were differentially expressed in PCR-negative contacts between pre-inoculation and 6 hr post-inoculation samples. Grey lines represent expression of genes in individual participants. Bold coloured lines indicate the median gene expression. Red lines = PCR-positive participants; blue lines = PCR-negative participants. Asterisks indicate significant differences in expression compared to pre-inoculation expression (Multiple Mann–Whitney U-test with Benjamini–Hochberg correction. ∗p<0.05, ∗∗p<0.01, ∗∗∗p<0.001.) PI = Pre-inoculation; 6 hr = 6 hours post-inoculation.

Supplemental Data Sheet

INSTINCT Study Investigators

Tables S1–S3 Table S1: **Environmental and behavioural contributors to transmission in PCR-positive and PCR-negative contacts.** Environmental swabs were collected from five frequently touched surfaces per household on study day 0. Swabs from the hands of household index cases were also collected on study day 0. PCR was performed on both environmental and hand swabs to detect SARS-CoV-2 contamination. Six behavioural changes of the contacts as a result of SARS-CoV-2 infection in households were documented using case record forms. All p values are from Fisher’s Exact Test comparing PCR-positive and PCR-negative contact responses. ∗PCR-positive households include all those in which 1 or more contacts became PCR-positive. PCR-negative households include all those in which all contacts remained persistently PCR-negative. Table S2: **Rooting study day samples to infection timepoints.** Samples from infected contacts were assigned to infection timepoints according to the study day on which the first PCR-positive nasopharyngeal sample was collected. Blood samples collected prior to PCR-detectable infection were denoted as Pre-PCR-Positive (PP) samples. Blood samples collected contemporaneous with the first PCR-positive nose and throat swab from a given contact were denoted as First PCR-Positive sample (FP) with subsequent samples being denoted as FP+n where n is the number of days after the first PCR-positive sample was taken. Table S3: **Exposure score elements stratified by PCR-positive, SC1 PCR-negative and SC2-4 PCR-negative contacts.** Relationship score describes the relationship of the contact to the index case with values of 100, 80, or 60 being given to contacts in romantic partnerships, parent/child or sibling relationships and other cohabitant relationships respectively. Room share score describes the level of room sharing throughout the house. Contacts who share a bedroom with the index case were given a score of 100, contacts which did not share a bedroom but did share a bathroom were given a score of 80, and a value of 60 was given to contacts who did not share a bedroom or bathroom but did share other living spaces (e.g., kitchen or living room) with the index case. p Value column displays results of Kruskal Wallis tests. Where significant differences were observed Dunn’s pairwise multiple comparison test with Bonferroni correction for multiple testing was performed. Both ‘Relationship Score’ and ‘Room Share Score’ were significantly higher in PCR-positive contacts compared to SC2-4 PCR-negative contacts (p = 0.0002 and p = 0.009 respectively). There was no significant difference in either metric between PCR-positive contacts and SC1 PCR-negative contacts.
