# Supplementary figures and images for: An ultra-early, transient interferon-associated innate immune response associates with protection from SARS-CoV-2 infection despite exposure
Source: eBioMedicine. 2024 Dec 11;111:105475. doi: 10.1016/j.ebiom.2024.105475 (PMC11697275; doi:10.1016/j.ebiom.2024.105475)

**Figure S1**

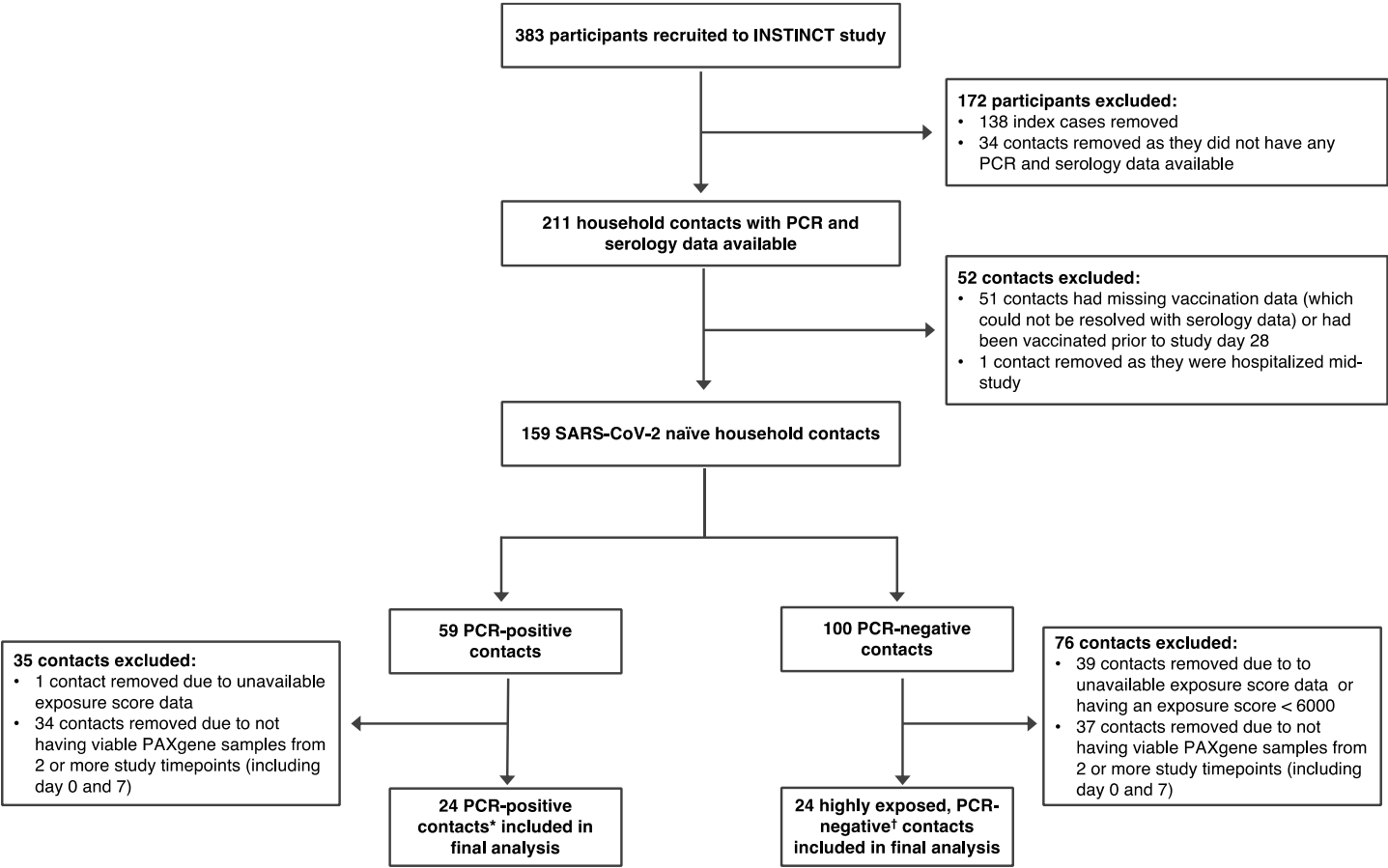

**Figure S2**

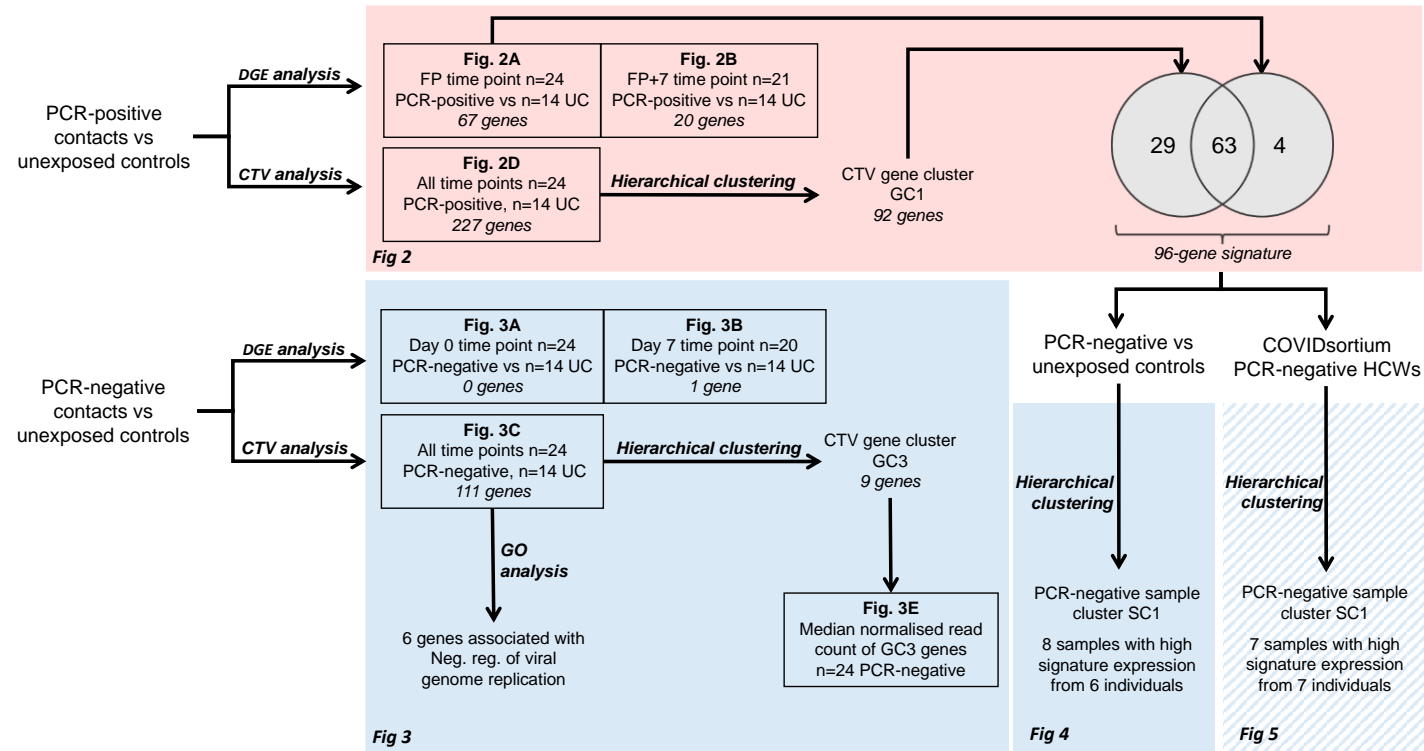

### Figure S3

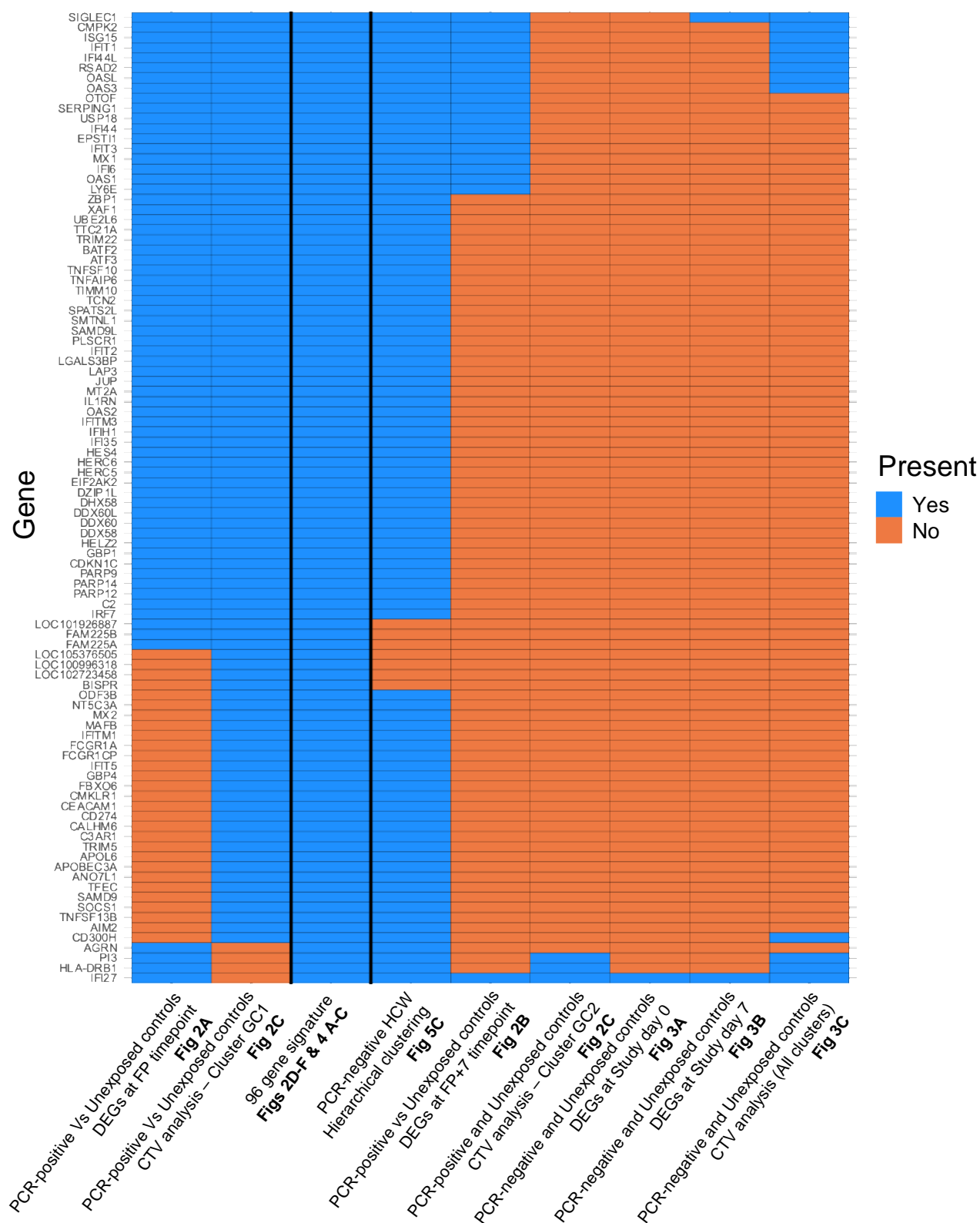

**Figure S4**

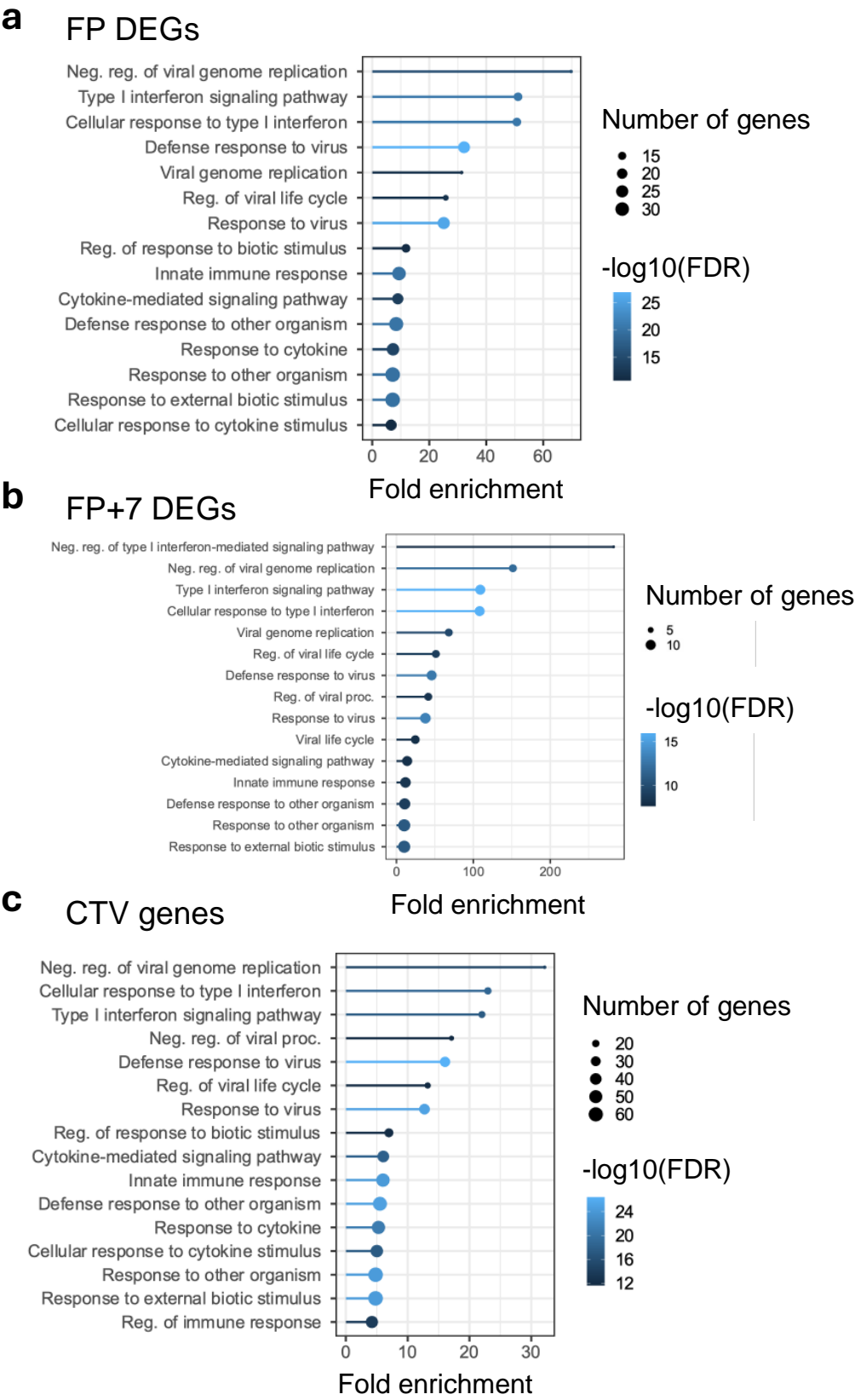

**Figure S5**

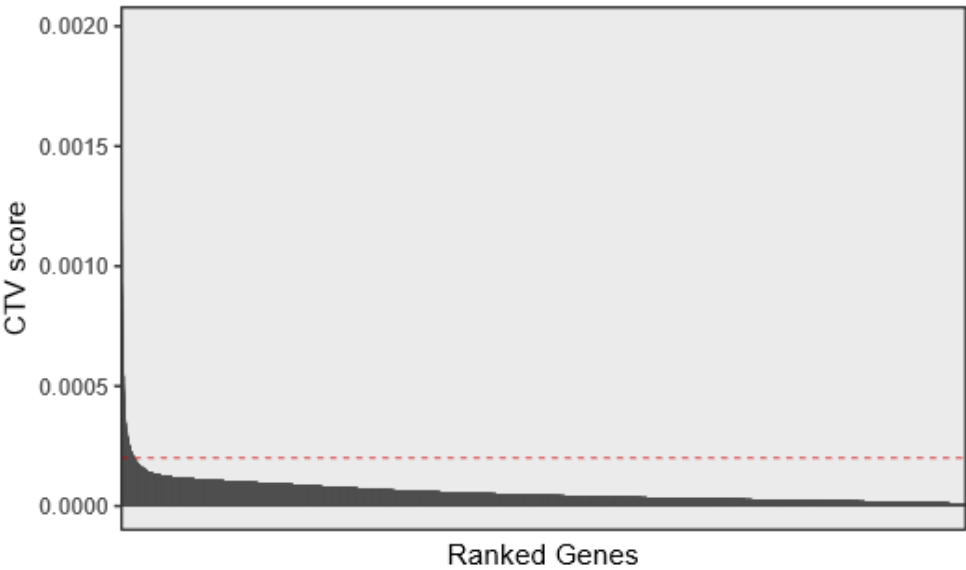

**Figure S6**

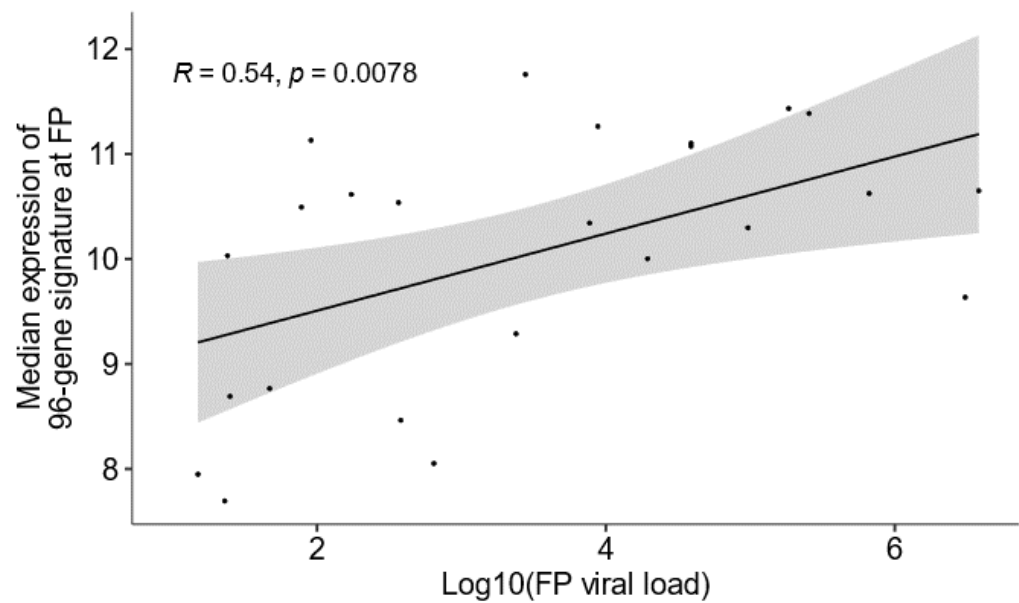

**Figure S7**

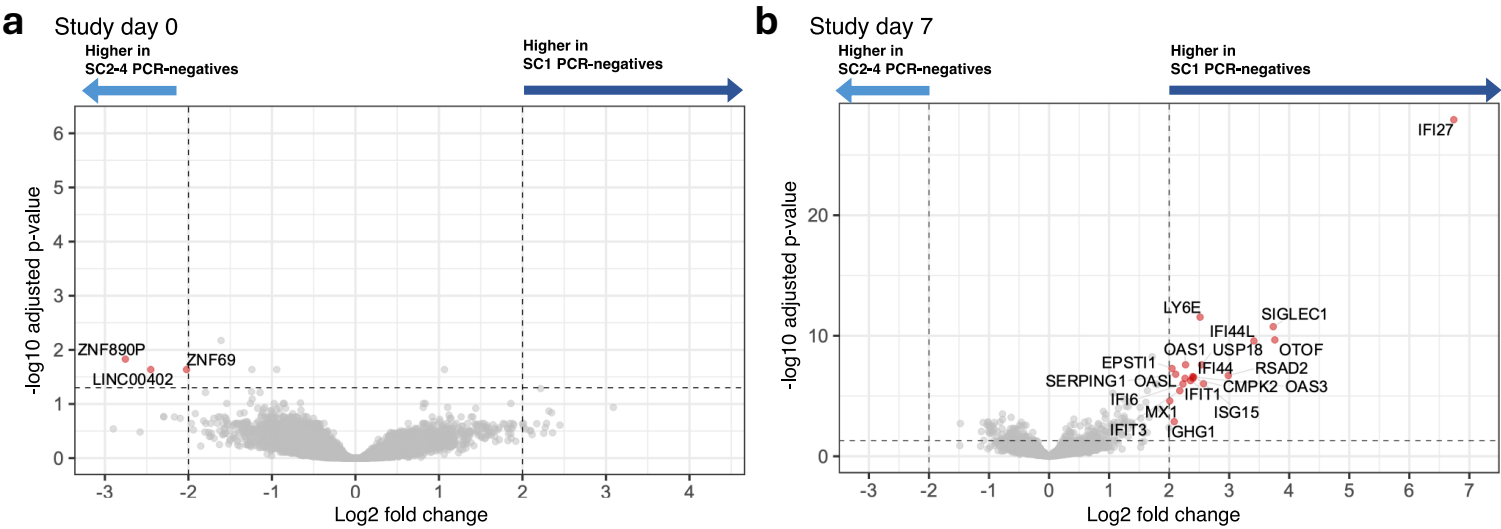

**Figure S8**

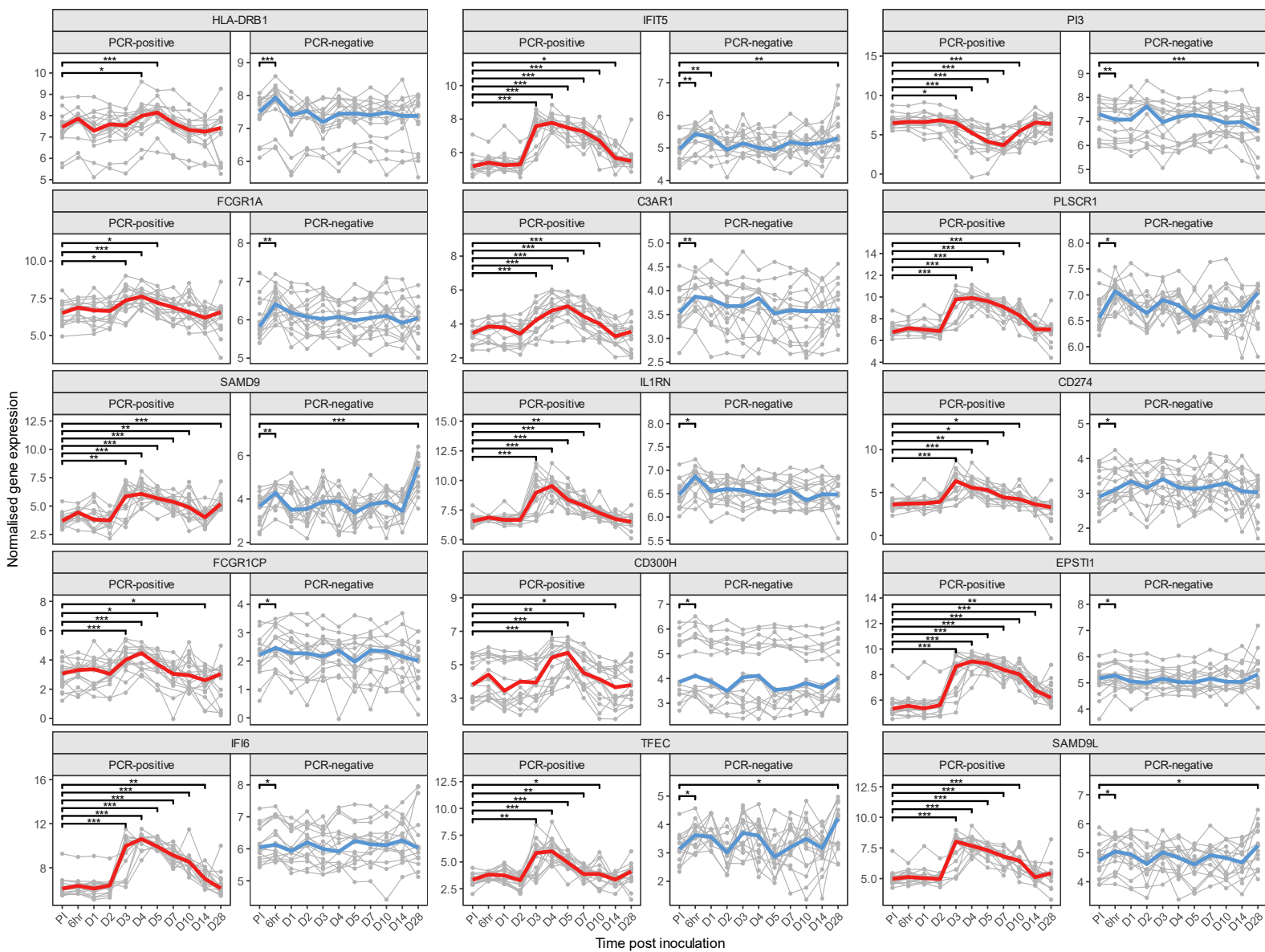

Supplement: Figures S1–S8 — Figure S1: Cohort inclusion criteria. Flowchart detailing the derivation of the cohort used in subsequent analyses from total INSTINCT study recruitment. ∗PCR-positive contact defined as having a PCR-positive result at 1 or more study timepoints. †PCR-negative contact defined as being persistently PCR-negative and seronegative at all timepoints with available data. Figure S2: Graphical representation of analytical pipeline. Type of analysis and groups of samples analysed for each figure are shown in separate, coloured boxes. FP = Time of First PCR-positive sample; FP+7 = 7 days post FP; UC = unexposed control; GC = gene cluster; SC = Sample cluster; HCW = Healthcare worker; CTV = Contribution to total variance; DGE = Differential gene expression; GO = Gene Ontology. Figure S3: Visualisation of overlap of gene lists. Heatmap indicating analyses in which genes comprising the 96-gene signature indicated in Fig. 3c were identified. Columns represent specific analyses presented in figures in the manuscript. Blue indicates where genes were identified, orange indicates where genes were not identified. FP = Time of First PCR-positive sample; FP+7 = 7 days post FP; DEG = differentially expressed genes; CTV = contribution to total variance; GC = gene cluster; HCW = healthcare worker. Figure S4: Gene Ontology (GO) analysis of gene lists derived from analyses of PCR-positive contacts vs unexposed controls. (a) Enriched GO terms for the 67 DEGs between FP samples from PCR-positive contacts and unexposed control samples depicted in Fig. 2a. (b) Enriched GO terms for the 20 DEGs between FP+7 samples from PCR-positive contacts and unexposed control samples depicted in Fig. 2b. (c) Enriched GO terms for the 227 high-variance genes identified from CTV analysis of all samples from PCR-positive contacts and unexposed controls depicted in Fig. 2d. All graphs produced using ShinyGo. FP = Time of First PCR-positive sample; FP+7 = 7 days post FP; DEG = differentially expressed genes; CTV = [file mmc1.pdf]
