# Supplementary material for: An ultra-early, transient interferon-associated innate immune response associates with protection from SARS-CoV-2 infection despite exposure
Source: eBioMedicine. 2024 Dec 11;111:105475. doi: 10.1016/j.ebiom.2024.105475 (PMC11697275; doi:10.1016/j.ebiom.2024.105475)
Supplement: INSTINCT Study Investigators [file mmc3.docx]

**INSTINCT Study Group Investigators:**

| **First names** | **Surnames** |
| --- | --- |
| Joe | Fenn |
| Kieran | Madon |
| Emily | Conibear |
| Romain | Derelle |
| Sean | Nevin |
| Rhia | Kundu |
| Seran | Hakki |
| Aleksandra | Koycheva |
| Nieves | Derqui |
| Mica | Tolosa-Wright |
| Jakob | Jonnerby |
| Lulu | Wang |
| Samuel | Baldwin |
| Timesh | Pillay |
| Ryan | Thwaites |
| Constanta | Luca |
| Robert | Varro |
| Anjna | Badhan |
| Eleanor | Parker |
| Carolina | Rosadas |
| Myra | McClure |
| Richard | Tedder |
| Graham | Taylor |
| Ajit | Lalvani |
| Janakan | Narean |
| Lucy | Mosscrop |
| Patricia | Watber |
| Jie | Zhou |
| Jack | Barnett |
| Hamish | Houston |
| Anika | Singanayagam |
| Paul | Freemont |
| Neil | Ferguson |
| Maria | Zambon |
| Wendy | Barclay |
| Jake | Dunning |
| Jessica | Cutajar |
| Valerie | Quinn |
| Sarah | Hammett |
| Eimèar | McDermott |
| Kristel | Timcang |
| Jada | Samuel |
| Samuel | Bremang |
| Samuel | Evetts |
| Megan | Davies |
| Chitra | Tejpal |
| Anjeli | Ketkar |
| Giulia | Miserocchi |
| Harriet | Catchpole |
| Simon | Dustan |
| Isaac | Day Weber |
| Federica | Marchesin |
| Alexandra | Kondratiuk |
