# Supplementary material for: An ultra-early, transient interferon-associated innate immune response associates with protection from SARS-CoV-2 infection despite exposure
Source: eBioMedicine. 2024 Dec 11;111:105475. doi: 10.1016/j.ebiom.2024.105475 (PMC11697275; doi:10.1016/j.ebiom.2024.105475)
Supplement: Tables S1–S3 — Table S1: Environmental and behavioural contributors to transmission in PCR-positive and PCR-negative contacts. Environmental swabs were collected from five frequently touched surfaces per household on study day 0. Swabs from the hands of household index cases were also collected on study day 0. PCR was performed on both environmental and hand swabs to detect SARS-CoV-2 contamination. Six behavioural changes of the contacts as a result of SARS-CoV-2 infection in households were documented using case record forms. All p values are from Fisher’s Exact Test comparing PCR-positive and PCR-negative contact responses. ∗PCR-positive households include all those in which 1 or more contacts became PCR-positive. PCR-negative households include all those in which all contacts remained persistently PCR-negative. Table S2: Rooting study day samples to infection timepoints. Samples from infected contacts were assigned to infection timepoints according to the study day on which the first PCR-positive nasopharyngeal sample was collected. Blood samples collected prior to PCR-detectable infection were denoted as Pre-PCR-Positive (PP) samples. Blood samples collected contemporaneous with the first PCR-positive nose and throat swab from a given contact were denoted as First PCR-Positive sample (FP) with subsequent samples being denoted as FP+n where n is the number of days after the first PCR-positive sample was taken. Table S3: Exposure score elements stratified by PCR-positive, SC1 PCR-negative and SC2-4 PCR-negative contacts. Relationship score describes the relationship of the contact to the index case with values of 100, 80, or 60 being given to contacts in romantic partnerships, parent/child or sibling relationships and other cohabitant relationships respectively. Room share score describes the level of room sharing throughout the house. Contacts who share a bedroom with the index case were given a score of 100, contacts which did not share a bedroom but did share a bathroom were [file mmc4.docx]

**SUPPLEMENTAL TABLES**

**Table S1**

| **Contributor to household transmission** | **Result** | **Total** | **PCR-**  **positive** | **PCR-**  **negative** | **P-value** |
| --- | --- | --- | --- | --- | --- |
| **Environmental swabs on Day 0 (per household)*** | Positive | 0/40 (0%) | 0/22 (0%) | 0/18 (0%) | 1 |
|  | Negative | 21/40 (52.5%) | 9/22 (40.9%) | 12/18 (66.7%) |  |
|  | Data not collected | 19/40 (47.5) | 13/22 (59.1%) | 6/18 (33.3%) |  |
|  |  |  |  |  |  |
| **Hand swab positivity of the index case on Day 0 (per household)*** | Positive | 6/40 (15.0%) | 6/22 (27.3%) | 0/18 (0%) | 0.06 |
|  | Negative | 29/40 (72.5%) | 15/22 (68.2%) | 14/18 (77.8%) |  |
|  | Data not collected | 5/40 (12.5%) | 1/22 (4.5%) | 4/18 (22.2%) |  |
|  |  |  |  |  |  |
| **Behavioral changes (per contact)** |  |  |  |  |  |
| **Changed bathrooms?** | Yes | 6/48 (12.5%) | 5/24 (20.8%) | 1/24 (4.2%) | 0.05 |
|  | No | 17/48 (35.4%) | 5/24 (20.8%) | 12/24 (50.0%) |  |
|  | Data not collected | 25/48 (52.1%) | 14/24 (58.4%) | 11/24 (45.8%) |  |
| **Changed bedrooms?** | Yes | 9/48 (18.7%) | 2/24 (8.3%) | 7/24 (29.2%) | 0.20 |
|  | No | 13/48 (27.1%) | 7/24 (29.2%) | 6/24 (25.0%) |  |
|  | Data not collected | 26/48 (54.2%) | 15/24 (62.5%) | 11/24 (45.8%) |  |
| **Changed other rooms?** | Yes | 7/48 (14.6%) | 4/24 (16.7%) | 3/24 (12.5%) | 0.65 |
|  | No | 16/48 (33.3%) | 6/24 (25.0%) | 10/24 (41.7%) |  |
|  | Data not collected | 25/48 (52.1%) | 14/24 (58.3%) | 11/24 (45.8%) |  |
| **Cleaned surfaces more?** | Yes | 26/48 (54.2%) | 11/24 (45.8%) | 15/24 (62.5%) | 0.05 |
|  | No | 13/48 (27.1%) | 7/24 (29.2%) | 6/24 (25.0%) |  |
|  | Data not collected | 9/48 (18.7%) | 6/24 (25.0%) | 3/24 (12.5%) |  |
| **Stayed 2 metres apart?** | Yes | 15/48 (31.3%) | 6/24 (25.0%) | 9/24 (37.5%) | 0.31 |
|  | No | 20/48 (41.6%) | 12/24 (50.0%) | 8/24 (33.3%) |  |
|  | Data not collected | 13/48 (27.1%) | 6/24 (25.0%) | 7/24 (29.2%) |  |
| **Washed hands more?** | Yes | 27/48 (56.3%) | 11/24 (45.8%) | 16/24 (66.7%) | 0.30 |
|  | No | 11/48 (22.9%) | 7/24 (29.2%) | 4/24 16.7%) |  |
|  | Data not collected | 10/48 (20.8%) | 6/24 (25.0%) | 4/24 (16.7%) |  |

**Table S2**

|  |  | **Infection timepoint** | | | | |
| --- | --- | --- | --- | --- | --- | --- |
| **Study Timepoint** |  | **PP** | **FP** | **FP+7** | **FP+14** | **Convalescent** |
|  | **Day 0** | 4 | 20 | 0 | 0 | 0 |
|  | **Day 7** | 0 | 4 | 18 | 0 | 0 |
|  | **Day 14** | 0 | 0 | 3 | 12 | 0 |
|  | **Day 28** | 0 | 0 | 0 | 0 | 7 |
|  | **Total** | 4 | 24 | 21 | 12 | 7 |

**Table S3**

| **Contributor to exposure score** | **Result** | **PCR-positive**  (N=23) | **SC1 PCR-**  **Negative**  (N=6) | **SC2-4 PCR-**  **Negative**  (N=18) | **P-value** |
| --- | --- | --- | --- | --- | --- |
| **Relationship score** | 100 | 17 (73.9%) | 3 (50%) | 3 (16.7%) | <0.0005 |
|  | 80 | 6 (26.1%) | 3 (50%) | 10 (55.5%) |  |
|  | 60 | 0 (0%) | 0 (0%) | 5 (27.8%) |  |
|  | Median (IQR) | 100 (90-100) | 90 (80-100) | 80 (65-80) |  |
|  |  |  |  |  |  |
| **Room share score** | 100 | 15 (65.2%) | 2 (33.3%) | 2 (11.1%) | <0.005 |
|  | 80 | 4 (17.4%) | 3 (50%) | 10 (55.6%) |  |
|  | 60 | 4 (17.4%) | 1 (16.7%) | 6 (33.3%) |  |
|  | Median (IQR) | 100 (80-100) | 80 (80-95) | 80 (60-80) |  |
|  |  |  |  |  |  |
| **Log (Index viral load AUC)** | Median  (IQR) | 4.35  (3.02-5.27) | 4.30  (4.13-5.59) | 2.91  (2.04-4.61) | 0.26 |
